# Azobenzene/Tetraethyl Ammonium Photochromic Potassium Channel Blockers: Scope and Limitations for Design of Para-Substituted Derivatives with Specific Absorption Band Maxima and Thermal Isomerization Rate

**DOI:** 10.3390/ijms222313171

**Published:** 2021-12-06

**Authors:** Daniil M. Strashkov, Vladimir N. Mironov, Dmitrii M. Nikolaev, Maxim S. Panov, Stanislav A. Linnik, Andrey S. Mereshchenko, Vladimir A. Kochemirovsky, Andrey V. Vasin, Mikhail N. Ryazantsev

**Affiliations:** 1 Institute of Biomedical Systems and Biotechnologies, Peter the Great St. Petersburg Polytechnic University, 29 Polytechnicheskaya Str., 195251 St. Petersburg, Russia; danielstr@mail.ru (D.M.S.); dmitrii.m.nikolaev@gmail.com (D.M.N.); linnik894@gmail.com (S.A.L.); lasergroupspb@gmail.com (V.A.K.); vasin_av@spbstu.ru (A.V.V.); 2 Nanotechnology Research and Education Centre RAS, Saint Petersburg Academic University, 8/3 Khlopina Street, 194021 St. Petersburg, Russia; vova_mironov_97@mail.ru; 3 Institute of Chemistry, Saint Petersburg State University, 7/9 Universitetskaya nab., 199034 St. Petersburg, Russia; m.s.panov@spbu.ru (M.S.P.); a.mereshchenko@spbu.ru (A.S.M.)

**Keywords:** photopharmacology, azobenzene, red-shifting azobenzenes, spectral tuning of azobenzene photoswitches, azobenzene thermal isomerization rate, photochromic ion channel blockers, DENAQ

## Abstract

Azobenzene/tetraethyl ammonium photochromic ligands (ATPLs) are photoactive compounds with a large variety of photopharmacological applications such as nociception control or vision restoration. Absorption band maximum and lifetime of the less stable isomer are important characteristics that determine the applicability of ATPLs. Substituents allow to adjust these characteristics in a range limited by the azobenzene/tetraethyl ammonium scaffold. The aim of the current study is to find the scope and limitations for the design of ATPLs with specific spectral and kinetic properties by introducing para substituents with different electronic effects. To perform this task we synthesized ATPLs with various electron acceptor and electron donor functional groups and studied their spectral and kinetic properties using flash photolysis and conventional spectroscopy techniques as well as quantum chemical modeling. As a result, we obtained diagrams that describe correlations between spectral and kinetic properties of ATPLs (absorption maxima of *E* and *Z* isomers of ATPLs, the thermal lifetime of their *Z* form) and both the electronic effect of substituents described by Hammett constants and structural parameters obtained from quantum chemical calculations. The provided results can be used for the design of ATPLs with properties that are optimal for photopharmacological applications.

## 1. Introduction

Photopharmacology is a fast-growing research area that is based on the conception of switching on/off the biological activity by the light stimulus [1,2,3,4,5,6]. Such processes become possible due to a structural reorganization that, after light absorption, occurs in the photoactive moiety and subsequently in the entire molecular system, changing its biological activity. The minimal required scaffold of a photopharmacological compound has to include both a photoactive part and a part that determines biological activity, e.g., ion channel blocking, signal transduction, etc. The development of an effective photopharmacological compound requires the adjustment of both optical and biological properties.

**Figure 1:**
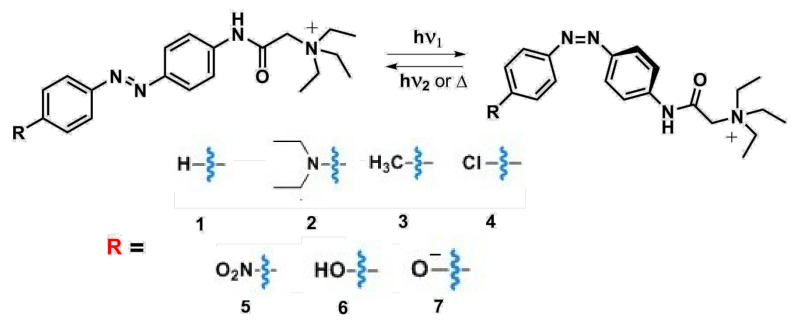
The structure of azobenzene/tetraethyl ammonium photochromic ligands considered in the current study.

The present study focuses on azobenzene/tetraethyl ammonium photochromic ligands (ATPLs, Figure 1) and aims to provide information that can be used for the rational design of ATPLs with specific absorption band maxima ($\lambda_{max}$) and thermal lifetime (τ) of less stable isomers. We limited the considered solvents to water and dimethyl sulfoxide (DMSO) as the most relevant solvents for photopharmacological applications. ATPLs belong to a well-known family of light-sensitive $K_{v}$ channel blockers that can be applied in a variety of contexts, such as nociception control or vision restoration [7,8,9,10,11,12,13]. Recently, ATPLs have been also used to control N-Methyl-d-aspartate receptors in rat brain neurons [14]. All ATPLs contain two common moieties: the photoactive azobenzene part and the tetraethyl ammonium group, conjugated to one of the azobenzene rings via an amide bond. ATPLs can undergo reversible photo or thermal isomerization from *E* to *Z* isomers and vice versa. Illumination by the light at the specific wavelength and sufficient intensity allows to achieve a photostationary equilibrium with the target concentration of one of the forms. The ratio of two forms at the photostationary state, the time required to achieve the photoequilibrium, and the relaxation time back to the thermodynamic equilibrium after switching the light off are important parameters that vary for different photopharmacological applications. These parameters, in turn, depend on the ratio of the extinction coefficients of two isomers at the chosen wavelength, quantum yields of photoisomerization for both isomers, relative energy of the isomers, and the thermal isomerization rate of the less stable isomer [15].

In the context of rational design of ATPLs with required properties, it is important to understand the scope and limits for adjusting ATPLs spectral and kinetic properties via modification of their structures. Generally, absorption band maxima of *E* and *Z* isomers of azobenzenes, as well as their thermal isomerization rates, can vary in quite a wide range [5]. However, for a minimal scaffold required to perform both photoswitching and biological functions, the variety is more limited. For ATPLs, the minimal possible scaffold, which is considered in this article, is compound **1** (Figure 1). The important feature of this scaffold is the presence of an electron-acceptor group at the para-position of one of the azobenzene rings. To keep a linear structure that is preferable for potassium channels blocking, the most common strategy to design ATPLs is to introduce another substituent at the para-position of the second azobenzene ring. These two factors, pre-existed electron-acceptor para-substituent and linear geometry, impose restrictions on the available pool of spectral properties and thermal isomerization rate of ATPLs. As demonstrated in a number of studies [16,17,18,19,20,21], $\lambda_{max}$ and τ of azobenzenes are determined by the electronic effect of a substituent (electron donor/electron acceptor) that can be quantified by Hammett constants [22] (σ) tabulated for many common substituents. Moreover, it is possible to evaluate correlation series such as $\lambda_{max}$/σ, τ/σ, etc., which can be used for further rational design. Investigation of scope and limitations for tuning ATPLs spectral and kinetic properties as well as evaluation of the above-mentioned correlations is the goal of the current study. To achieve this goal, a number of derivatives of **1** with different substituents at the para-position (Figure 1) have been synthesized, and their spectral and kinetic properties in water and DMSO have been studied by means of flash photolysis and conventional spectroscopy. The tabulated Hammett constants (σ) of substituents, as well as structural parameters of ATPLs obtained from ab initio quantum chemical calculations, have been used as variables to plot correlation diagrams.

## 2. Results

### 2.1. Spectral Properties of ATPLs

**Figure 2:**
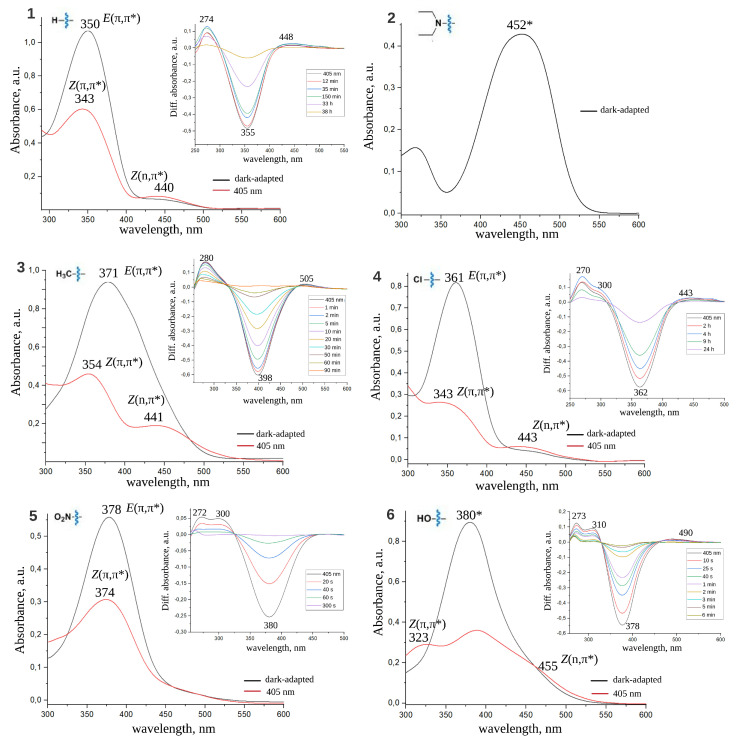
Dark-adapted and light-adapted spectra of investigated ATPLs in DMSO. Transient absorption UV-Vis spectra are shown in the insets. Spectral bands are related to *E*, *Z* isomers and ($\pi ,\pi^{*}$), ($n,\pi^{*}$) transitions. * For compounds **2** and **6** the band can be decomposed into two Gaussian functions (see Supplementary Materials for details).

**Figure 3:**
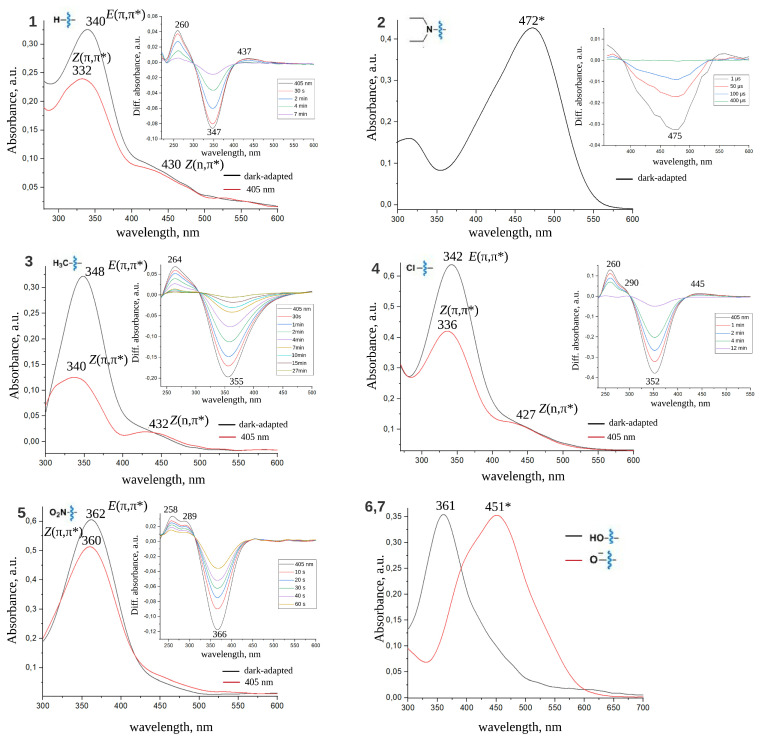
Dark-adapted and light-adapted spectra of investigated ATPLs in water. Transient absorption UV-Vis spectra are shown in the insets. Spectral bands are related to *E*, *Z* isomers and ($\pi ,\pi^{*}$), ($n,\pi^{*}$) transitions. * For compounds **2** and **7** the band can be decomposed into two Gaussian functions (see Supplementary Materials for details).

Figure 2 and Figure 3 show dark-adapted spectra for all investigated compounds and light-adapted spectra recorded after 30 s illumination with 405 nm or 532 nm light ($P_{405\mathrm{nm}}$ = 2 mW/mm${}^{2}$; $P_{532\mathrm{nm}}$ = 1 mW/mm${}^{2}$) for compounds that isomerize back to the stable form slowly enough to achieve photostationary equilibrium under given light intensity within 30 s. Transient UV-vis absorption spectra for all compounds are given in insets of these figures (for details of the used experimental setup see “Materials and Methods” section). All spectral bands are interpreted in the terms of *E* and *Z* isomers and ($n,\pi^{*}$) and ($\pi ,\pi^{*}$) transitions. Absorption maxima of all bands are specified. $\lambda_{max}$ for *E* and *Z* isomers of all considered compounds are also compiled in Table 1. For compounds **2** and **6** in DMSO absorption bands at 452 and 380 nm, respectively, can not be fitted by a single Gaussian function and requires two Gaussians. Similarly, for compounds **2** (471 nm) and **7** (451 nm) in water absorption bands exhibit complex structure and can be properly fitted by two Gaussian functions. The Gaussian maxima found from the Gaussian decomposition described above are interpreted as $\lambda_{max}$ for ($n,\pi^{*}$) and ($\pi ,\pi^{*}$) transitions and given in Table 1 (see Supplementary Materials for details).

From the perspective of photopharmacological applications of ATPLs two points are the most important: to shift the absorption band to the visible region of spectra and further to the infrared region, and/or to separate *Z* and *E* absorption bands as much as possible. Spectra of *Z* and *E* azobenzenes exhibit two bands: an intensive S${}_{0}$→ S${}_{2}$ ($\pi ,\pi^{*}$) band and a red-shifted weak S${}_{0}$→ S${}_{1}$ ($n,\pi^{*}$) band [5]. As a rule, the ($\pi ,\pi^{*}$) band of *E* isomer is more intensive and red-shifted compared to the ($\pi ,\pi^{*}$) band of *Z* isomer. On the contrary, the ($n,\pi^{*}$) band of *Z* isomer is more intensive than the ($n,\pi^{*}$) band of *E* isomer but both of them are much weaker than ($\pi ,\pi^{*}$) bands. To shift the photoequilibrium to the required isomer one can use two light sources that correspond to the best possible separation of *E* and *Z* bands. Two most common strategies utilize either ($\pi ,\pi^{*}$) bands for both isomers or the ($\pi ,\pi^{*}$) band of the *E* isomer and the ($n,\pi^{*}$) band of the *Z* isomer.

**Figure 4:**
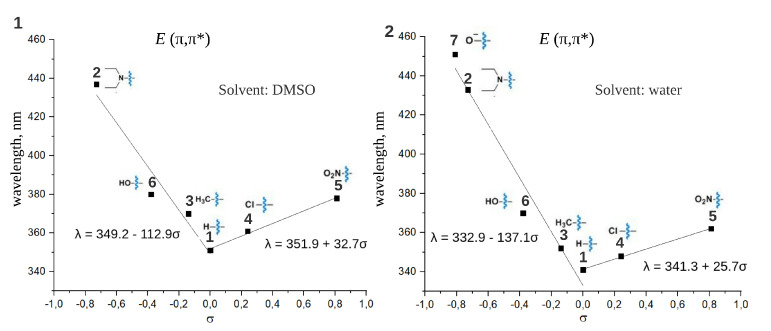
The dependence of absorption band maximum of the *E* form ($\lambda_{max}^{E}$) for the ($\pi ,\pi^{*}$) transitions on the Hammett constants of substituents (σ). Spectra are recorded in DMSO (**1**) and water (**2**). The dependencies were approximated by linear functions for σ > 0 and σ < 0 separately, parameters of approximations are given in the figure.

Figure 4 (1),(2) represents the correlation between $\lambda_{max}^{E}$ for the ($\pi ,\pi^{*}$) transition and Hammett constants for spectra recorded in DMSO and water, respectively. $\lambda_{max}$ ($\pi ,\pi^{*}$) are similar for both solvents and range from 340 nm for substance **1** in water to 451 nm for substance **7** in water. Both electron donor and electron acceptor substituents red-shift $\lambda_{max}^{E}$ ($\pi ,\pi^{*}$) but to a different extent. Adding an electron donor group (σ<0) to the parent compound **1** leads to so-called ”push-pull” compounds that are known both for a significant red shift of ($\pi ,\pi^{*}$) band and the acceleration of thermal isomerization rate of *Z* isomer. The limit $\lambda_{max}^{E}$ ($\pi ,\pi^{*}$) = 451 nm has been found for compound **7** with strong electron donor substituent (σ=−0.81). The $\lambda_{max}^{E}$ ($\pi ,\pi^{*}$) red shift caused by electron acceptor substituents (σ>0) is not so prominent and reaches only 378 nm for substance **5** (σ=0.81) in water. The compound **7** with a very strong electron donor substituent $-O^{-}$ (σ=−0.81) is mostly of theoretical interest and exists only in basic solutions. Apparently, compound **2**, DENAQ, which is already widely used in photopharmacology [10,24,25], provides the red shift of the $\lambda_{max}^{E}$ ($\pi ,\pi^{*}$) band that is close to the limits with the given scaffold, a single para-substituent, and at physiological pH.

**Figure 5:**
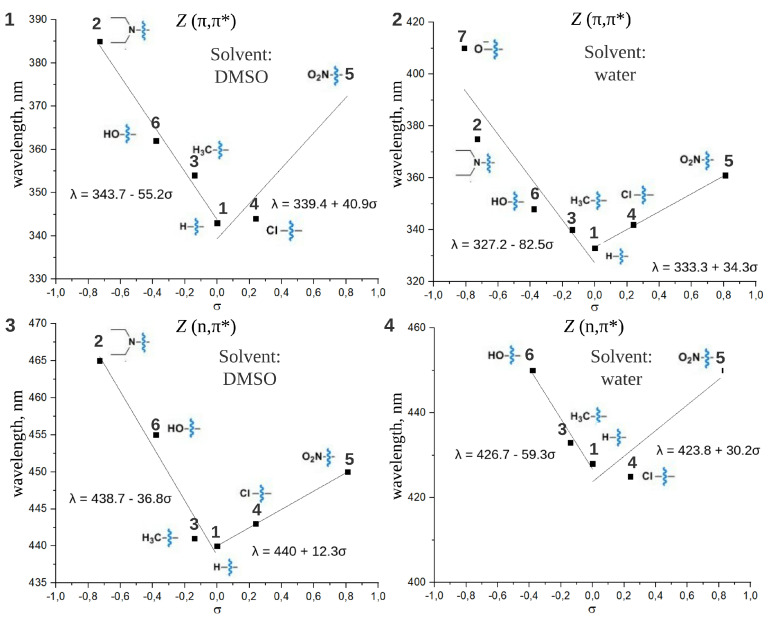
The dependence of absorption band maxima of the *Z* form ($\lambda_{max}^{Z}$) for the ($\pi ,\pi^{*}$) and ($n,\pi^{*}$) transitions on the Hammett constants of substituents (σ). Spectra are recorded in DMSO (**1**,**3**) and water (**2**,**4**). The dependencies were approximated by linear functions for σ > 0 and σ < 0 separately, parameters of approximations are given in the figure.

**Figure 6:**
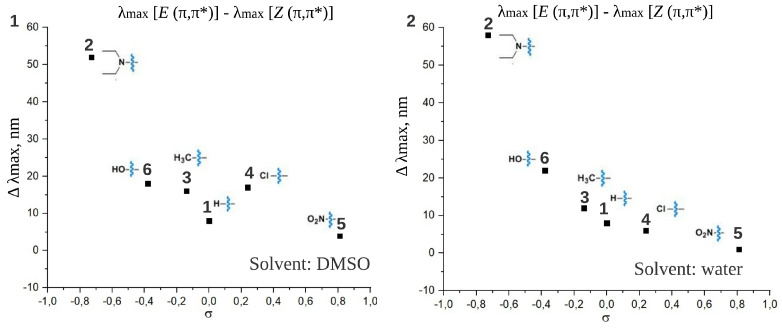
The dependence of spectral shift between absorption band maxima of *E* and *Z* forms of ATPLs ($\Delta \lambda_{max}=\lambda_{max}^{E}$ - $\lambda_{max}^{Z}$) for the ($\pi ,\pi^{*}$) transitions on the Hammett constants of substituents (σ) in DMSO (**1**) and water (**2**).

The similar V-shaped trends are found for maxima of both ($\pi ,\pi^{*}$) and ($n,\pi^{*}$) absorption bands for the *Z* form of the studied compounds (Figure 5). All $\lambda_{max}^{Z}$ ($\pi ,\pi^{*}$) are blue-shifted comparing to $\lambda_{max}^{E}$ ($\pi ,\pi^{*}$). Figure 6(1),(2) presents the separation of *E* ($\pi ,\pi^{*}$) and *Z* ($\pi ,\pi^{*}$) bands in DMSO and water, respectively. For compound **2** with the most negative σ in the given set, the difference between $\lambda_{max}^{E}$ ($\pi ,\pi^{*}$) and $\lambda_{max}^{Z}$ ($\pi ,\pi^{*}$) is 58 nm and 52 nm in DMSO and water, respectively. This difference becomes smaller with increasing σ and reaches only several nm for the compound **5** with the most positive σ in the set. Therefore, the better ($\pi ,\pi^{*}$) band separation, which is a very important condition for photopharmacological applications, can be achieved via introducing an electron donor group. On the contrary, the introduction of an electron acceptor group impedes the separation of ($\pi ,\pi^{*}$) bands. For the majority of considered compounds (see $\lambda_{max}$ given in Table 1) the separation between maxima of *E* ($\pi ,\pi^{*}$) and *Z* ($n,\pi^{*}$) spectral bands does not depend significantly on the studied substituents and is about 80 nm in both solvents. The only exception is the “push-pull” compound **2**, DENAQ, where this difference is only 28 nm in DMSO [10].

**Figure 7:**
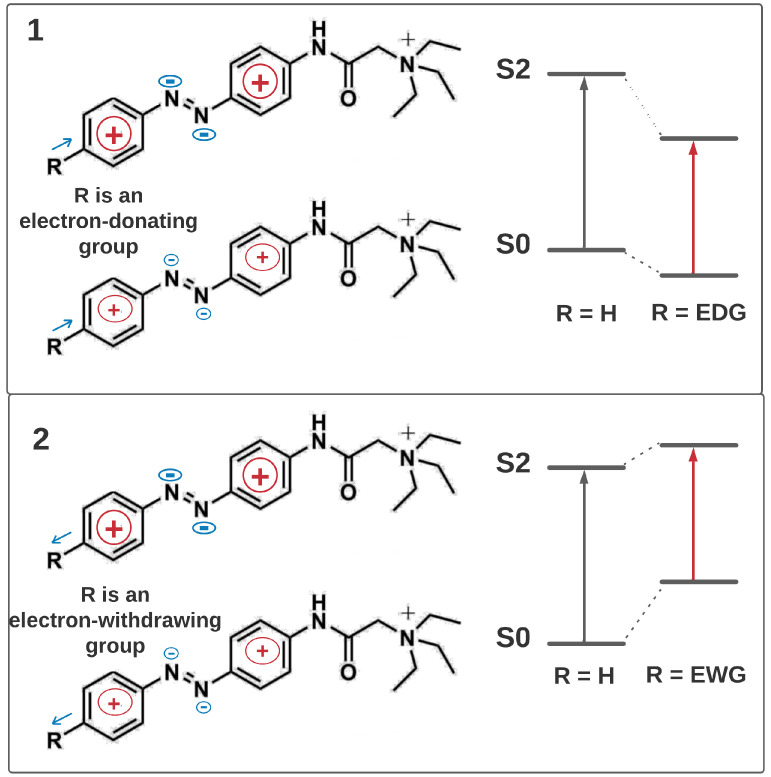
The effect of the charge transfer on the absorption maximum of the considered ATPLs.

**Figure 8:**
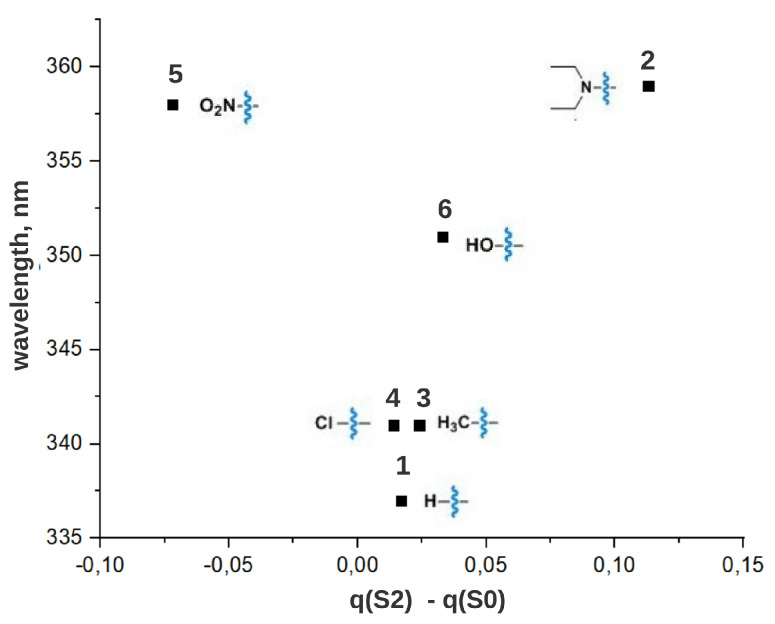
The dependence of $\lambda_{max}^{Z}$ $\left(\pi ,\pi^{*}\right)$ on the charge value relocated from/to a substituent after excitation in the considered ATPLs.

$\lambda_{max}$ for all compounds calculated at the TD-CAM-B3LYP-D/6-31g*//B3LYP-D/6-31g* (PCM = water, DMSO) level of theory are given in Supplementary Materials. The calculated $\lambda_{max}$ values are in reasonable agreement with experimental data with a systematic shift around 30 nm for $\lambda_{max}^{E}$$\left(\pi ,\pi^{*}\right)$, around 50 nm for $\lambda_{max}^{Z}$$\left(\pi ,\pi^{*}\right)$, around 15 nm for $\lambda_{max}^{Z}$$\left(n,\pi^{*}\right)$. To rationalize the V-shape of the correlation plots described above we calculated Mulliken charges both for ground states S${}_{0}$ and for the second excited states S${}_{2}$ for all compounds considered in the article in water. The calculations were performed at B3LYP-D/6-31g* (PCM=water) and TD-B3LYP-D/6-31g*//B3LYP-D/6-31g* (PCM=water) level of theory, respectively. Analysis of the charge distribution before and after S${}_{0}$→ S${}_{2}$ transition for compound **1** reveals that the electron density (negative charge) relocates from the benzene rings to the azo-group of the compound (see Figure 7 and Figure S4b in the Supplementary Materials for additional details). Analysis of the same electron density difference for the rest of the compounds (Figure S4c–g in Supplementary Materials) suggests an explanation, as depicted in Figure 7. Electron donating groups stabilize both S${}_{0}$ and S${}_{2}$ states but at different extents. Larger stabilization of S${}_{2}$ compared to S${}_{0}$ leads to a red shift of the corresponding band. Similarly, the difference in destabilization of these two states after introducing the electron withdrawing group results again in a red shift. In addition, Figure 8 demonstrates that the dependence of $\lambda_{max}^{Z}$$\left(\pi ,\pi^{*}\right)$ on the charge value relocated from/to a substituent after excitation exhibits a similar V-shaped plot. A similar mechanism of tuning has also been reported for a number of compounds, i.e., for photosensitive proteins rhodopsins [26,27,28], and apparently governs solvatochromism of azobenzene derivatives in polar solvents [29].

### 2.2. Thermal Isomerizaion Rate of ATPLs *Z* Isomers

**Figure 9:**
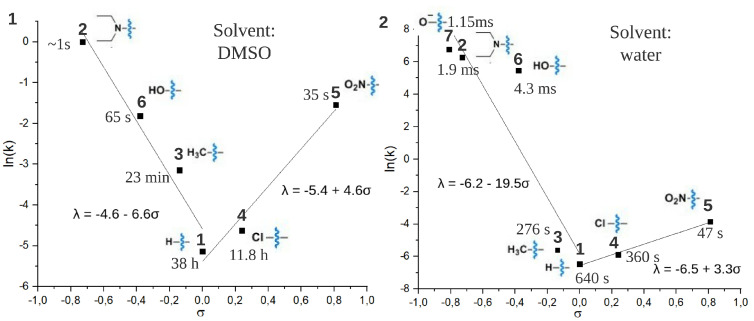
The dependence of the logarithm of thermal isomerization kinetic constant of *Z* ATPLs (ln(k)) on the Hammett constants of substituents (σ) in DMSO (**1**) and water (**2**). The values of thermal lifetimes of *Z* forms are also given in the figure. The dependencies were approximated by linear functions for σ > 0 and σ < 0 separately. Parameters of approximations are given in the figure.

For all compounds considered in this study, the *Z* isomer is thermodynamically less stable than the *E* isomer. Figure 9(1),(2) represents the dependence ln(k)/σ for the *Z* form of all investigated compounds in DMSO and water, respectively. Because in the water solution the *Z* form of compounds **2**, **6**, **7** isomerizes back to the *E* form on the subpicosecond time scale, a flash photolysis technique was applied to evaluate the lifetime (τ) of this decay. The lifetime for the rest of the compounds varies from seconds to hours. The measured τ are shown in Figure 9(1),(2) and also compiled in Table 2. The *Z* isomer of parent compound **1** exhibits the slowest decay back to *E* isomer both in DMSO (τ=38 h) and water (τ=640 s). Similar to the correlations found for absorption maxima and Hammett substituent constants, ln(k)/σ correlation plots exhibit non-symmetrical V-shape curvature imposing the upper limit on τ of ATPLs. The effect of electron donor substituents (σ<0) is again more prominent than the effect of electron acceptor substituents (σ>0). In DMSO, as well as in other aprotic solvents, the V-shape of the correlation plot can be rationalized by a change in mechanism for the thermal cis-trans isomerization from rotation to inversion [30]. In water the isomerization for substituents with σ<0 occurs much faster than in DMSO and falls in the microsecond range for compounds **2**, **6**. This acceleration can be explained by the presence of a non-negligible concentration of azonium cation at physiological pH [31,32,33], which also depends on the electronic effects of the substituents and/or by hydrogen-bond-catalyzed changes in the thermal isomerization pathway [34,35,36]. Additional investigations are necessary to clarify this point. When increasing σ, the difference of lifetime in water and DMSO decreases and becomes negligible for the -NO${}_{2}$ substituent (compound **5**).

**Figure 10:**
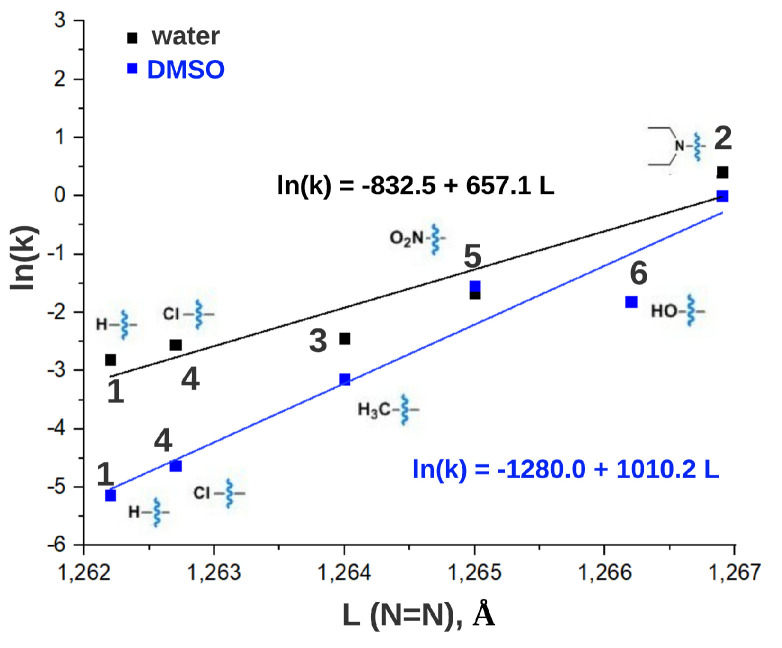
The dependence of the logarithm of thermal isomerization kinetic constants of *Z* ATPLs (ln(k)) on the length of the double bond (in Å) of the azobenzene moiety in *E* ATPLs.

Figure 10 demonstrates that correlation also exists between a thermal lifetime of *Z* isomer and structural parameters of *E* isomer obtained with quantum chemical calculations. Specifically, a linear correlation was found between the logarithm of thermal isomerization kinetic constant (ln(k)) and the length of the double bond of the azobenzene moiety of the *E* isomer.

### 2.3. Influence of the Tetraethyl Ammonium Group on Spectral and Kinetic Properties

**Figure 11:**
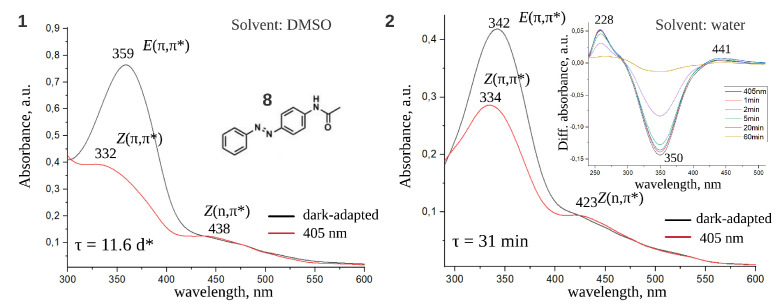
Dark-adapted and light-adapted spectra of compound **8** in DMSO (**1**) and water (**2**). Transient absorption UV-Vis spectrum in water is shown in the inset. Spectral bands are related to *E*, *Z* isomers and ($\pi ,\pi^{*}$), ($n,\pi^{*}$) transitions. * Thermal lifetime of *E* isomer in DMSO from the work [11].

We have also investigated spectral and kinetic properties of the compound **8** (Figure 11, Table 1 and Table 2). For **8** dissolved in DMSO $\lambda_{max}$ and τ have already been reported in the recent study [11]. Our results confirmed the data from this work and provided additional data for **8** dissolved in water. Compound **8** differs from compound **1** (Figure 1), which is taken as the scaffold in the current study, by the tetraethyl ammonium group connected to acetyl substituent. Although this modification affects $\lambda_{max}$ only slightly, it does significantly change τ both in DMSO and water (Table 1 and Table 2), stressing the importance of the proper choice of scaffold for screening.

## 3. Materials and Methods

### 3.1. Synthesis and Purification

All commercially available reagents were purchased from Sigma-Aldrich, abcr GmbH, TCI, Carl Roth GmbH, and used without further purification. Column chromatography was performed on silica gel 60 M (0.04–0.063 mm) and C18 silica gel 0.035–0.07 mm. All organic solvents were anhydrous. To synthesize ATPLs we used two previously published protocols [8,37], the details can be found in the Supporting Information.

### 3.2. UV-Vis Spectra

Electronic absorption spectra were measured using two-beam scanning spectrophotometer Lambda 1050 equipped with a double monochromator. These measurements were performed in a range between 300 and 600 nm with a spectral resolution of 2 nm. UV/Vis spectroscopy with second-to-hour time resolution was performed on NanoDrop 2000 using a quartz glass cuvette (10 mm pathlength), light for switching was delivered by a CoolLED pE-4000 ($P_{405\mathrm{nm}}$ = 2 mW/mm${}^{2}$; $P_{532\mathrm{nm}}$ = 1 mW/mm${}^{2}$). Results were exported and plotted in Origin 9.0 software, and kinetic traces were fitted monoexponentially using expdecay1.

### 3.3. Flash Photolysis Technique

Microsecond transient absorption (ΔA) spectra and kinetics were obtained using two setups for flash photolysis based on Nd:YAG (Surelite-I, Continuum, 5 ns FWHM, 20 mJ per pulse) laser with the second harmonic at 532 nm for **2** and the third harmonic at 355 nm for **6** the sources of the flash photoexcitation. The photodynamics was monitored by continuous exposure to a tungsten halogen light source (AvaLight-HAL, Avantes) and a xenon arc lamp (150 W) for **2** and **6**, respectively, used as probe lights. The ΔA signal was detected by a silicon photoreceiver (2051-FS, New Focus) and a photomultiplier tube (Hamamatsu R-2949) for **2** and **6**, respectively, and recorded with a TDS3032B (Tektronix, 300 MHz) digitizing oscilloscope. For all time-resolved experiments ΔA spectra were recorded in the wavelength range of 350–600 nm. The pump and probe lights were transmitted through the 1 cm quartz cuvette with the sample solution perpendicularly to each other. Each sample solution was bubbled with Ar at an ambient temperature of 25 ${}^{\circ }$C for at least 20 min prior to the measurements. The ΔA kinetic traces as a function of delay time (t) were fitted to a sum of exponential functions using Origin 9.0 software: $\Delta A=\Delta A_{0}+\sum \Delta A_{i}e^{-t/\tau_{i}}$, where $\tau_{i}$ the time constant, $\Delta A_{i}$ is the amplitude, $\Delta A_{0}$ is the permanent spectrum at infinitely long times (>>1 ns).

### 3.4. Quantum Chemical Calculations

Geometry optimization for all studied compounds was performed at B3LYP-D/6-31g* (PCM = water, DMSO) level of theory [38]. Spectral properties were calculated on optimized geometry with TD-CAM-B3LYP/6-31g* (PCM = water, DMSO) methodology. Gaussian09 program package was used for all calculations [39].

## 4. Conclusions

Drug design in pharmacology in general, and photopharmacology in particular, requires scanning libraries of candidates to optimize several physicochemical and biochemical parameters at the same time. The design of photopharmacological compounds requires the investigation of an additional dimension of photochemical properties. Techniques that are already widely used in drug design, such as scaffold-based design and structure/properties correlations, can be implemented also in photopharmacology research [40]. Descriptors that are commonly used in drug design, such as Hammett constants or structural parameters, can be also utilized to tune spectral and kinetic properties. In this study, we take this approach to investigate the scope and limitations for tuning spectral and kinetic properties of azobenzene/tetraethyl ammonium scaffold (Figure 1), which is widely used as the framework for the design of photoactive potassium channel blockers. Substituents were chosen to span a wide range of Hammett constants from strong electron donors to strong electron acceptors. Although this study focuses on the effects of para-substituents, the obtained plots can be used also for meta-substituents. Ortho-substituents require special attention due to the possible additional electronic and steric interactions, which are not properly described by Hammett constants.

For the scaffold investigated in the current study, the red-shift of $\lambda_{max}^{E}$ ($\pi ,\pi^{*}$) can be achieved via introduction of both the electron donor substituent (σ<0) and the electron acceptor substituent (σ>0), with the effect of the donor substituents being more pronounced. However, both of these approaches have a drawback. On one hand, σ decreasing for donor substituents leads to an increase of the thermal lifetime of *Z* isomer and, consequently, to difficulties with obtaining the required concentration of the *Z* isomer at the photostationary state. This decrease in lifetime is especially prominent if water is used as the solvent and is less significant for DMSO. On the other hand, σ increasing for acceptor substituents leads to a larger overlap of the *Z* and *E* ($\pi ,\pi^{*}$) bands and again to difficulties with the population of the less stable *Z* form. The separation and relative intensity of the *Z* and *E* ($n,\pi^{*}$) bands as well as their separations with the *E* ($n,\pi^{*}$) band is not affected significantly by substituents and deviates slightly from numbers reported for the azobenzene (see [5] for a review).

In some cases these drawbacks are not critical and azobenzene/tetraethyl ammonium photochromic ligands have already found many applications. However, if there is a necessity to go beside the above-described limits, a modification of the present scaffold in its azobenzene part is apparently the most practical way to do it. Several modifications can be considered: extension of the π-electron system by introducing additional benzene rings or other π-conjugated parts; switching to tetra-ortho-substituted azobenzenes [41,42,43], bridged azobenzenes (dihydrobenzodiazocines) [44], azo heteroarene scaffolds [45], or even to use other known classes of photoswitches.

## Figures and Tables

**Table 1:** Absorption band maxima of ATPLs considered in the current study. For compounds **2** and **6** in DMSO and compounds **2** and **7** in water the absorption band was decomposed into two Gaussian functions (see Supplementary Materials for details). σ is the Hammett constant of substituents. $\lambda_{max}^{E}$ ($\pi ,\pi^{*}$) and $\lambda_{max}^{Z}$ ($\pi ,\pi^{*}$) are the absorption band maxima of the ($\pi ,\pi^{*}$) transition of the *E* and *Z* isomers, respectively. $\lambda_{max}^{Z}$ ($n,\pi^{*}$) is the absorption band maximum for the ($n,\pi^{*}$) transition of the *Z* isomer.

Compound	σ [23]	$\lambda_{\max}^{E}$ ($\pi ,\pi^{*}$) Water, nm	$\lambda_{\max}^{E}$ ($\pi ,\pi^{*}$) DMSO, nm	$\lambda_{\max}^{Z}$ ($\pi ,\pi^{*}$) Water, nm	$\lambda_{\max}^{Z}$ ($\pi ,\pi^{*}$) DMSO, nm	$\lambda_{\max}^{Z}$ ($n,\pi^{*}$) Water, nm	$\lambda_{\max}^{Z}$ ($n,\pi^{*}$) DMSO, nm
**1**	0.00	340	350	332	343	430	440
**2**	−0.72	433	437	-	385 [10]	-	465 [10]
**3**	−0.14	348	371	340	354	432	441
**4**	0.24	342	361	336	343	427	443
**5**	0.81	362	378	360	374	-	-
**6**	−0.37	370	380	348	362	450	455
**7**	−0.81	451	-	410	-	-	-
**8**	-	342	359	334	332	423	438

**Table 2:** The lifetime of *Z* isomers (τ) of ATPLs considered in the current study in DMSO and water. σ is the Hammett constant of substituents.

Compound	σ [23]	τ (DMSO)	τ (Water, PBS)
**1**	0.00	38 h	640 s (pH = 7.4)
**2**	−0.72	≈1 s	1.9 ms (pH = 7.4)
**3**	−0.14	23 min	276 s (pH = 7.4)
**4**	0.24	11.8 h	360 s (pH = 7.4)
**5**	0.81	35 s	47 s (pH = 7.4)
**6**	−0.37	65 s	4.3 ms (pH = 7.4)
**7**	−0.81	-	1.15 ms (pH = 11.3)
**8**	-	11.6 d [11]	31 min (pH = 7.4)

## Supplementary Materials

The supplementary materials are available online at https://www.mdpi.com/article/10.3390/ijms222313171/s1.
