# Supplementary material for: Azobenzene/Tetraethyl Ammonium Photochromic Potassium Channel Blockers: Scope and Limitations for Design of Para-Substituted Derivatives with Specific Absorption Band Maxima and Thermal Isomerization Rate
Source: Int J Mol Sci. 2021 Dec 6;22(23):13171. doi: 10.3390/ijms222313171 (PMC8658355; doi:10.3390/ijms222313171)
Supplement: Supplementary file 1 [file ijms-22-13171-s001.zip › ijms-1460040-supplementary.pdf]

Supplementary information for the manuscript
"Azobenzene/tetraethyl ammonium photochromic potassium channel blockers: scope and limitations for design of para-substituted derivatives with specific absorption band maxima and thermal isomerization rate"

Daniil M. Strashkov, Vladimir N. Mironov, Dmitrii M. Nikolaev, Maxim S. Panov, Stanislav A. Linnik, Andrey S. Mereshchenko, Vladimir A. Kochemirovsky, Andrey V. Vasin, Mikhail N. Ryazantsev

1. Calculated absorption maxima values.

Table S1. Calculated absorption maxima values of ATPLs in water (method TD CAM-B3LYP/6-31g*, PCM(SCRF) for water, DMSO).

In water solution:

Substituent	λmax trans (π,π*)	λmax trans (n,π*)	λmax cis (π,π*)	λmax cis (n,π*)
**1**	337	451	287	460
**2**	359	448	301	467
**3**	341	447	289	464
**4**	341	451	290	462
**5**	358	470	361	468
**6**	351	441	294	466
**7**	412	420	334	453

in DMSO solution:

Substituent	λmax trans (π,π*)	λmax trans (n,π*)
**1**	337	451
**2**	361	448
**3**	342	448
**4**	343	452

**5**	360	471
**6**	352	441
**7**	412	421

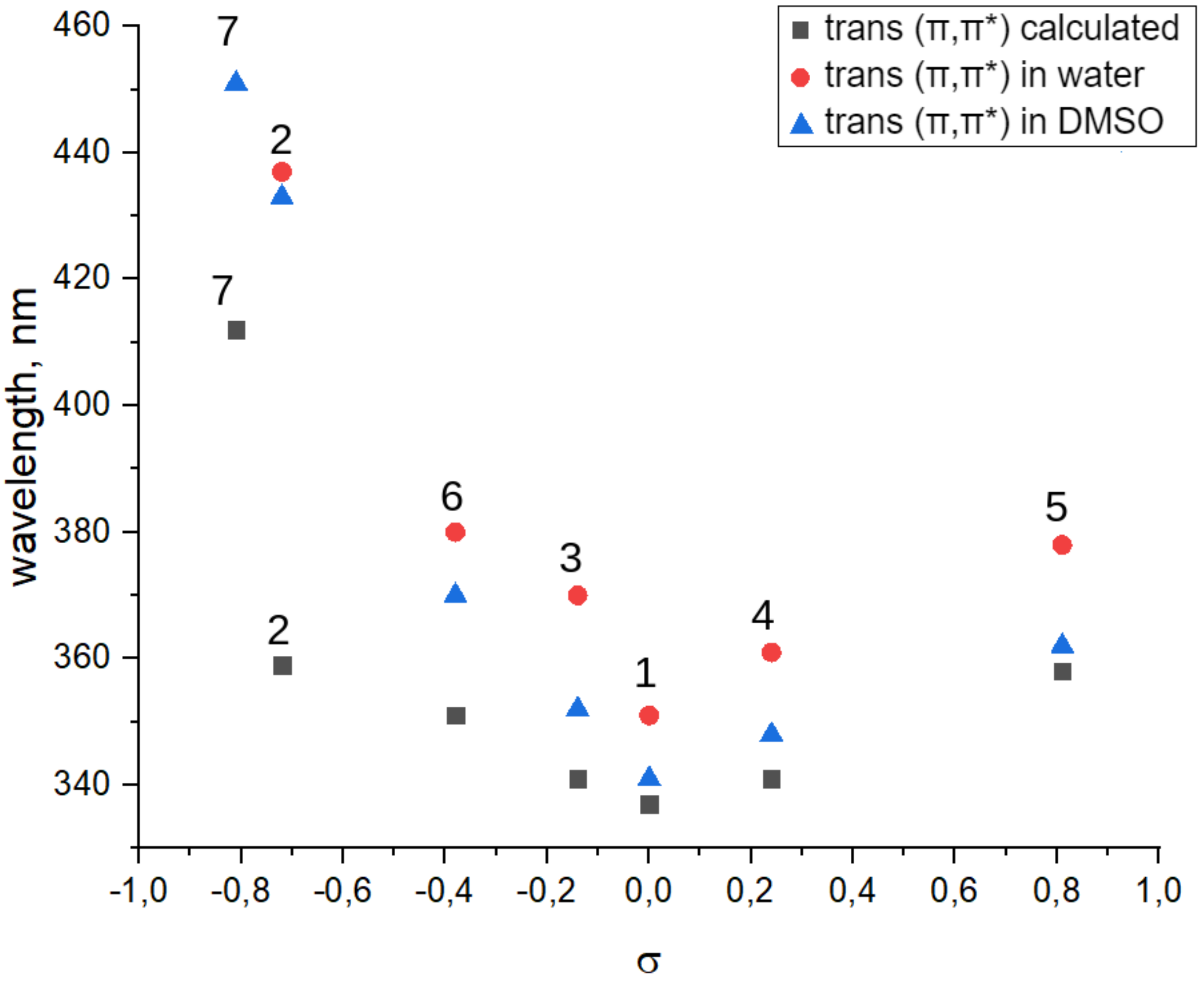

Figure S1. Experimental and calculated λ_{max} values for (π,π*) transition of ATPLs in the trans form as a function of Hammett constant of substituents.

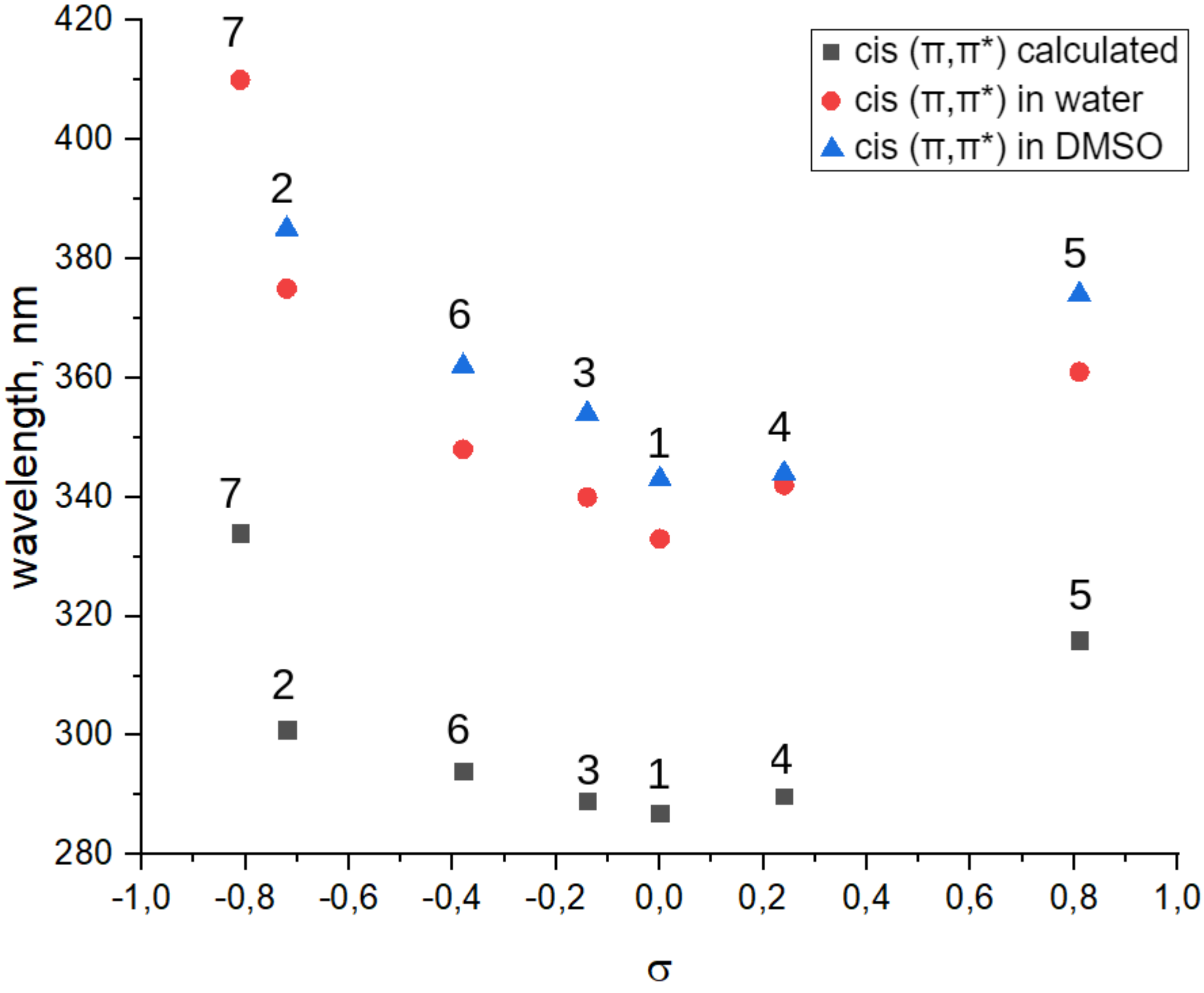

Figure S2. Experimental and calculated λ_{max} values for (π,π*) transition of ATPLs in the cis form as a function of Hammett constant of substituents.

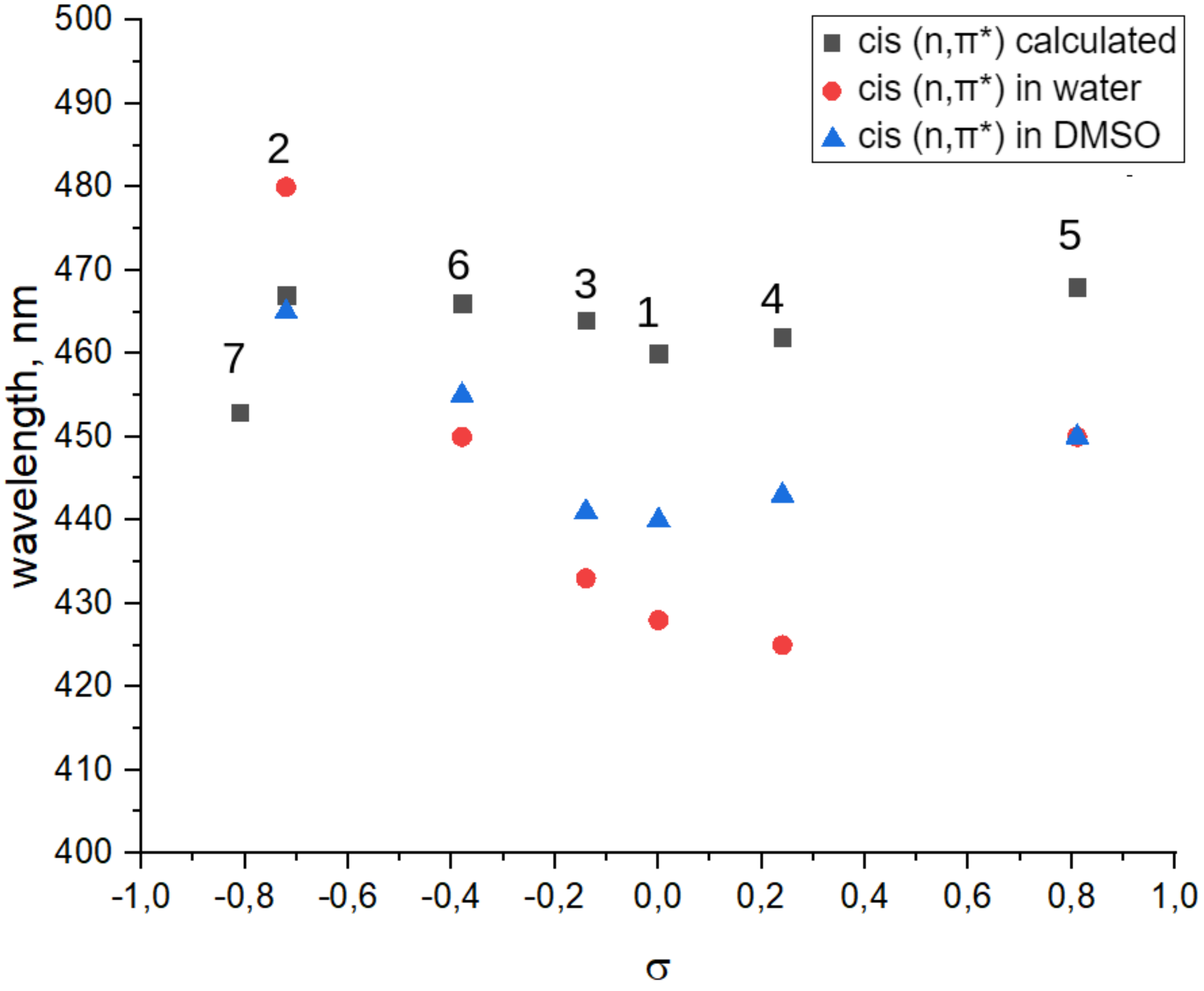

Figure S3. Experimental and calculated λ_{max} values for (n,π*) transition of ATPLs in the cis form as a function of Hammett constant of substituents.

2. Calculated Mulliken atomic charges of considered compounds.

a) Azobenzene:

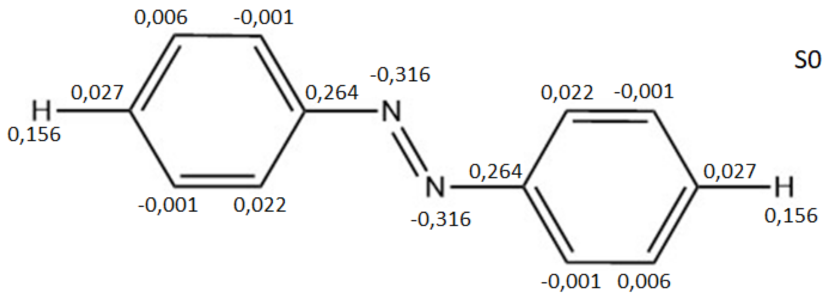

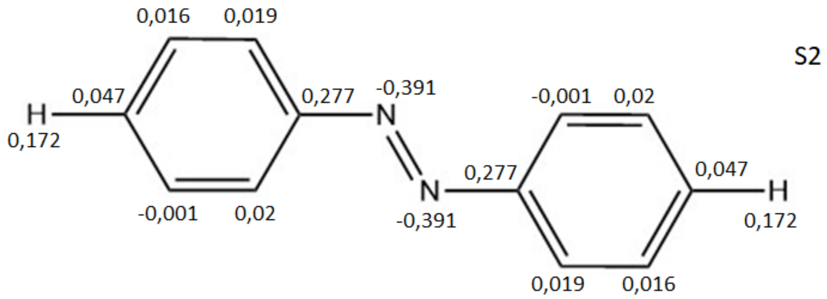

b) Compound 1:

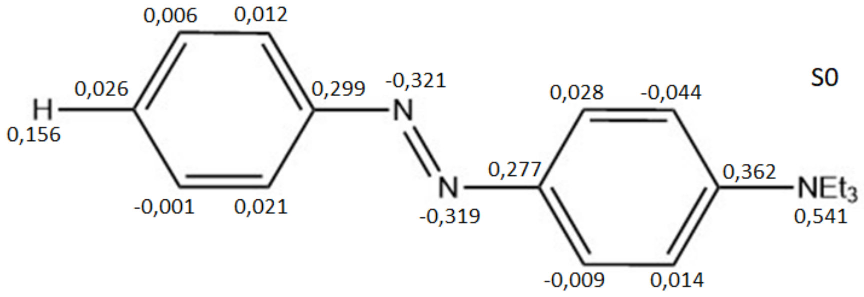

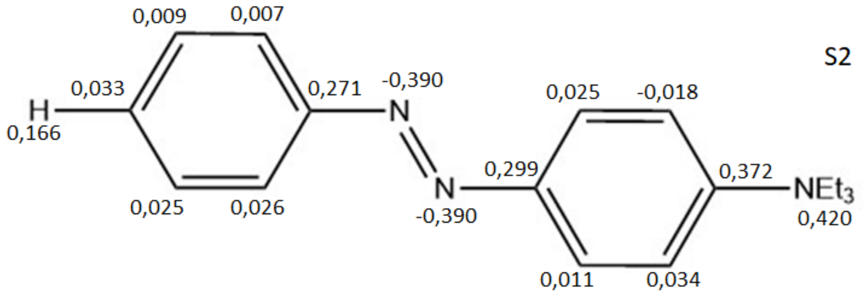

c) Compound 2 (DENAQ):

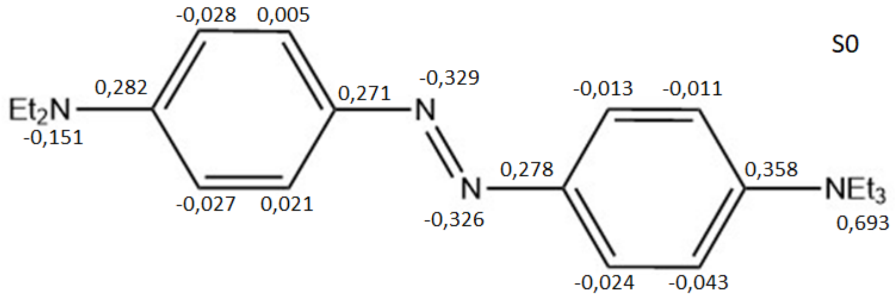

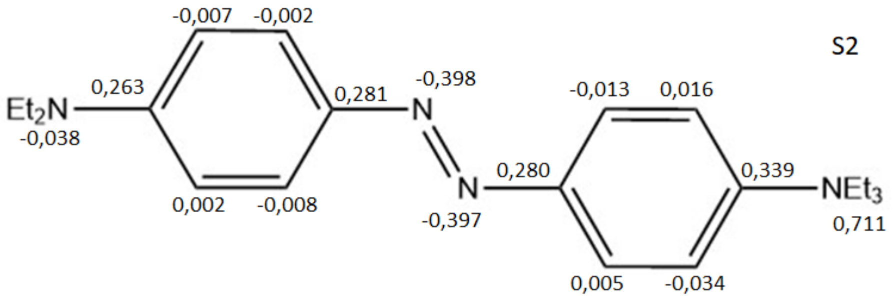

d) Compound 3:

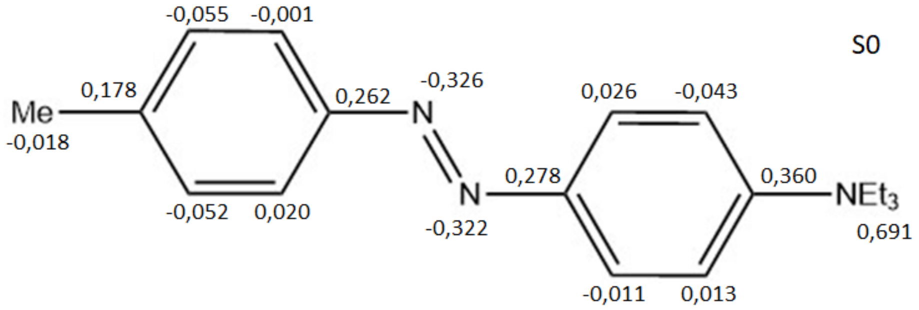

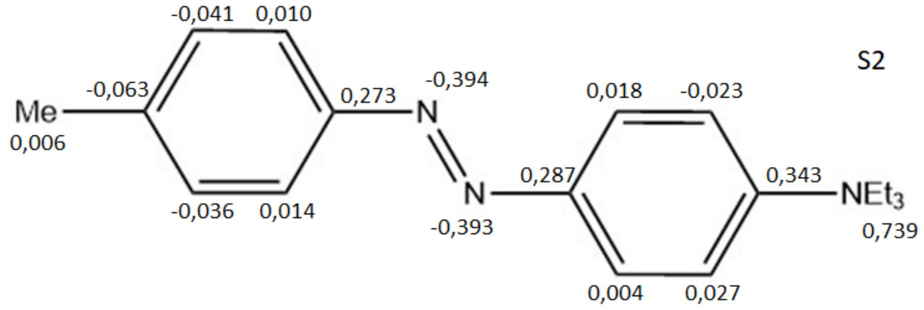

e) Compound 4:

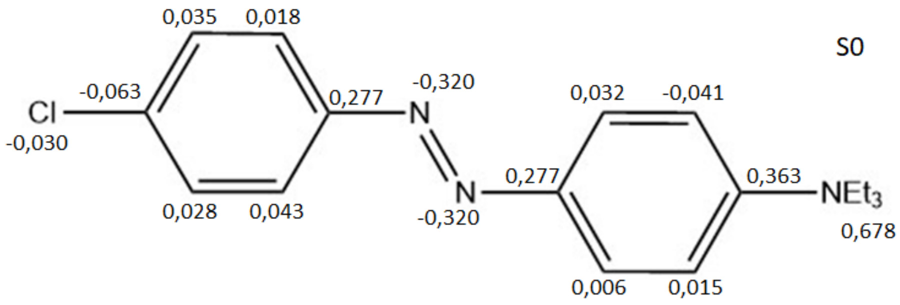

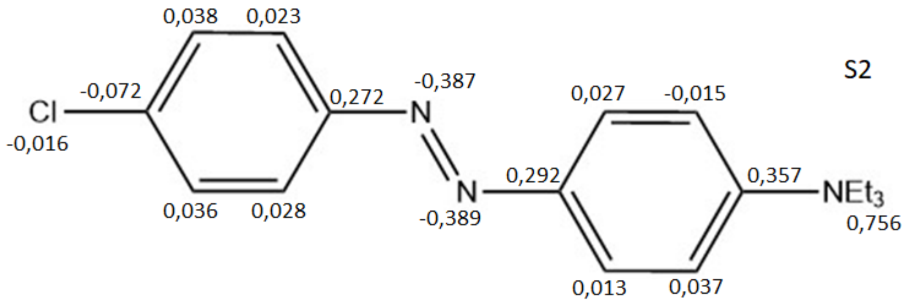

f) Compound 5:

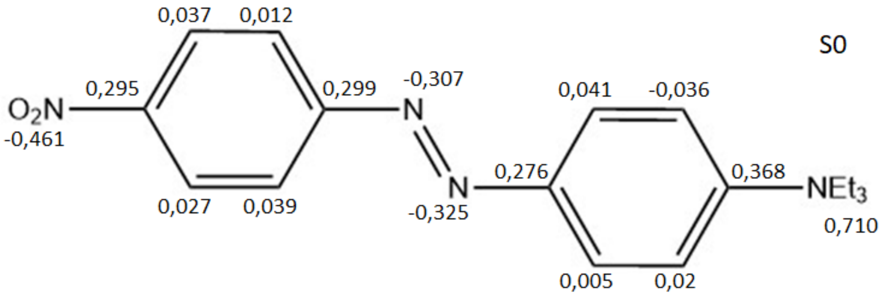

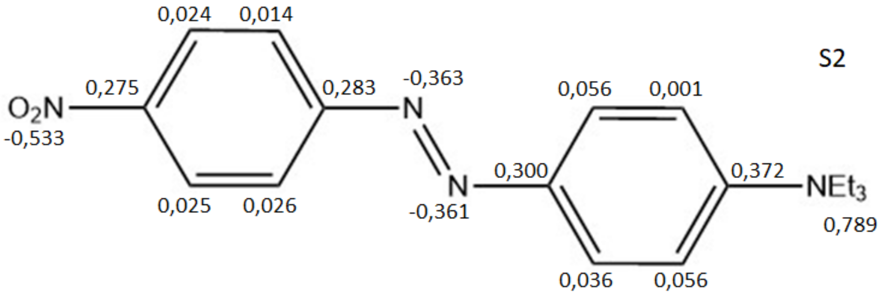

g) Compound 6:

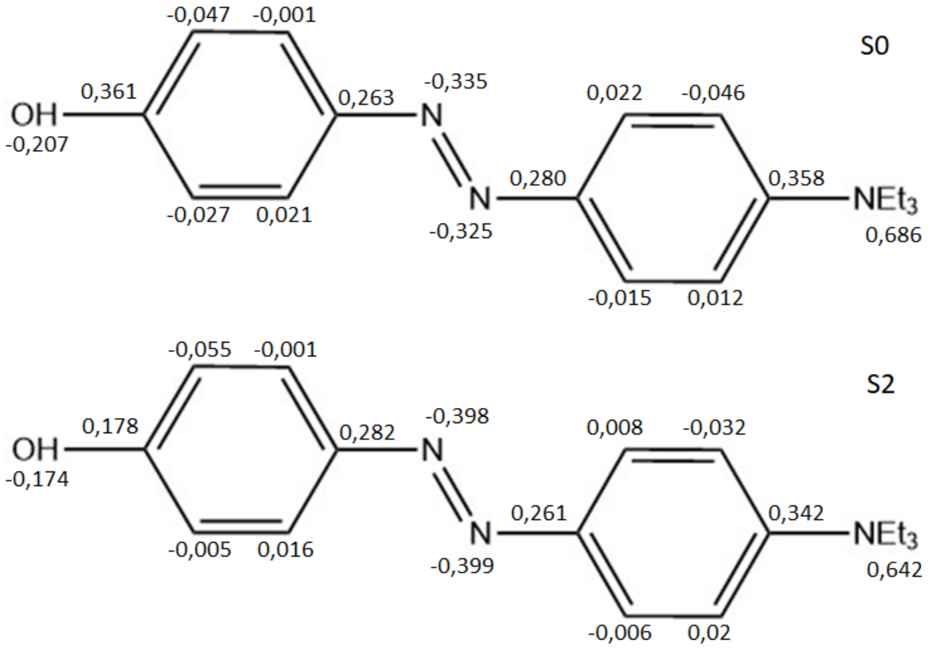

Figure S4. Calculated Mulliken atomic charges of considered compounds.

3. Derivation of the cis form concentration in the photostationary state.

To derive the concentration of cis form in photostationary equilibrium as a function of Hammett constant of substituents we used the linear dependence of the logarithm of the thermal isomerization kinetic constant on Hammett constant ln(k_thermo)(σ) (Figure 9), and the equation that describes the change of cis form concentration with time. Here we neglect trans to cis backward thermal isomerisation.

$$\frac{dC_{cis}}{dt} = -k_{therm}C_{cis} + k_{photo}(1 - C_{cis})QY_{photoisom} \quad (1)$$

where k_therm is the thermal isomerisation kinetic constant

$$k_{therm} = \frac{1}{\tau_{therm}} \quad (2)$$

k_photo is the number of photons absorbed by molecules of ATPL in one second.

$$k_{photo} = \frac{1}{\tau_{photo}} = \frac{N_{abs}}{\nu N_a} \quad (3)$$

The photostationary equilibrium is described by the following equation:

$$\frac{C_{cis}}{C_{trans}} = \frac{k_{photo}}{k_{therm}} QY_{photoisom} \quad (4)$$

The kinetic constant for reaching photostationary equilibrium can be expressed as:

$$k_{PSS} = k_{photo} QY_{photoisom} + k_{therm} \quad (5)$$

For small OD values the following equation is valid:

$$\frac{I - I_0}{I_0} = 1 - e^{-\epsilon l C} \approx \epsilon l C = 2.3OD \quad (6)$$

Then we can derive k_photo as a function of the extinction coefficient:

$$k_{photo} = \frac{P\lambda\epsilon}{\hbar c N_a} \quad (7)$$

or as a function of OD value:

$$k_{photo} = \frac{2.3P\lambda OD}{Cl\hbar c N_a} \quad (8)$$

Here P is power density (W/m^2), *l* is the optical path length, C is molar concentration, QY is the photoisomerisation quantum yield, Na is the Avogadro number, λ is the illumination wavelength, c is the speed of light, ħ is the Planck's constant.

Using Equations 4, 8 and the approximation for the ln(k)(σ) obtained in the current study (Figure 9), we derived the concentration of cis form in photostationary state as a function of Hammett constant. Here we assume that the laser illuminates only the trans form of the compound, laser power density is ~1mW/mm^2 and OD value is 0.1 at the maximum absorption wavelength (405 nm) (Figure S5).

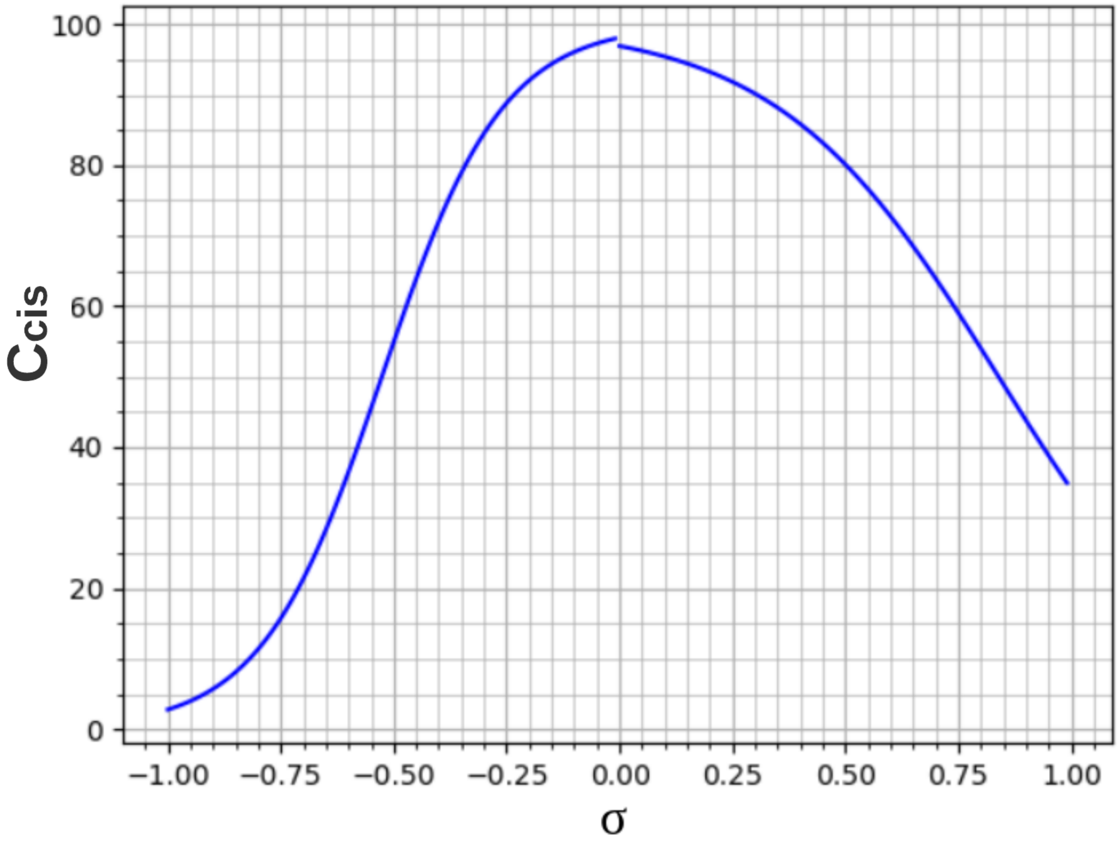

Figure S5. Dependence of the cis form concentration in photostationary state on the Hammett constant.

4. Decomposition of absorption bands of compounds 2, 6, 7 into Gaussian functions.

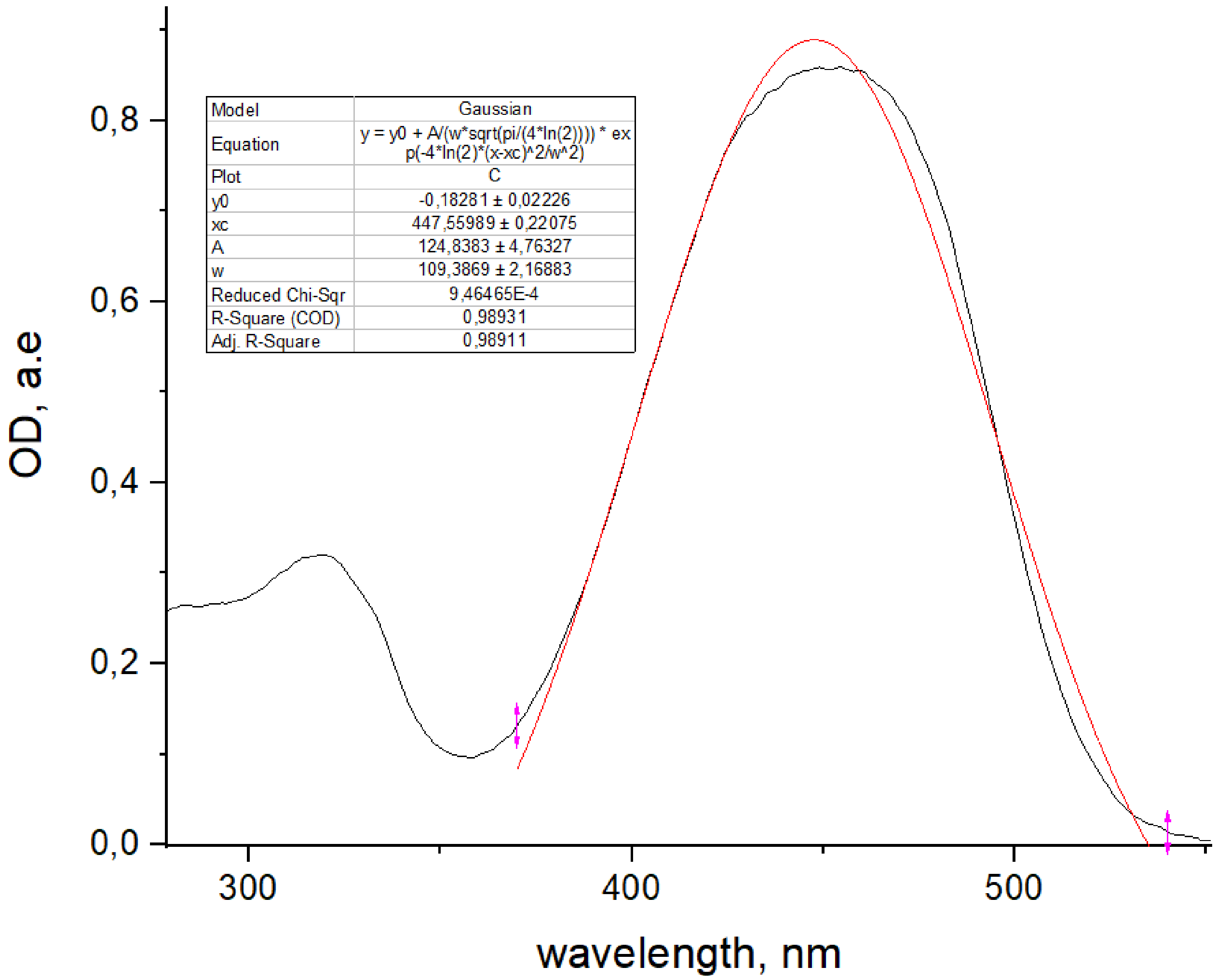

Figure S6. Approximation of the absorption band of compound **2** (DENAQ) in DMSO by a single Gaussian function. The presented result shows that the curve is poorly approximated by a single Gaussian function.

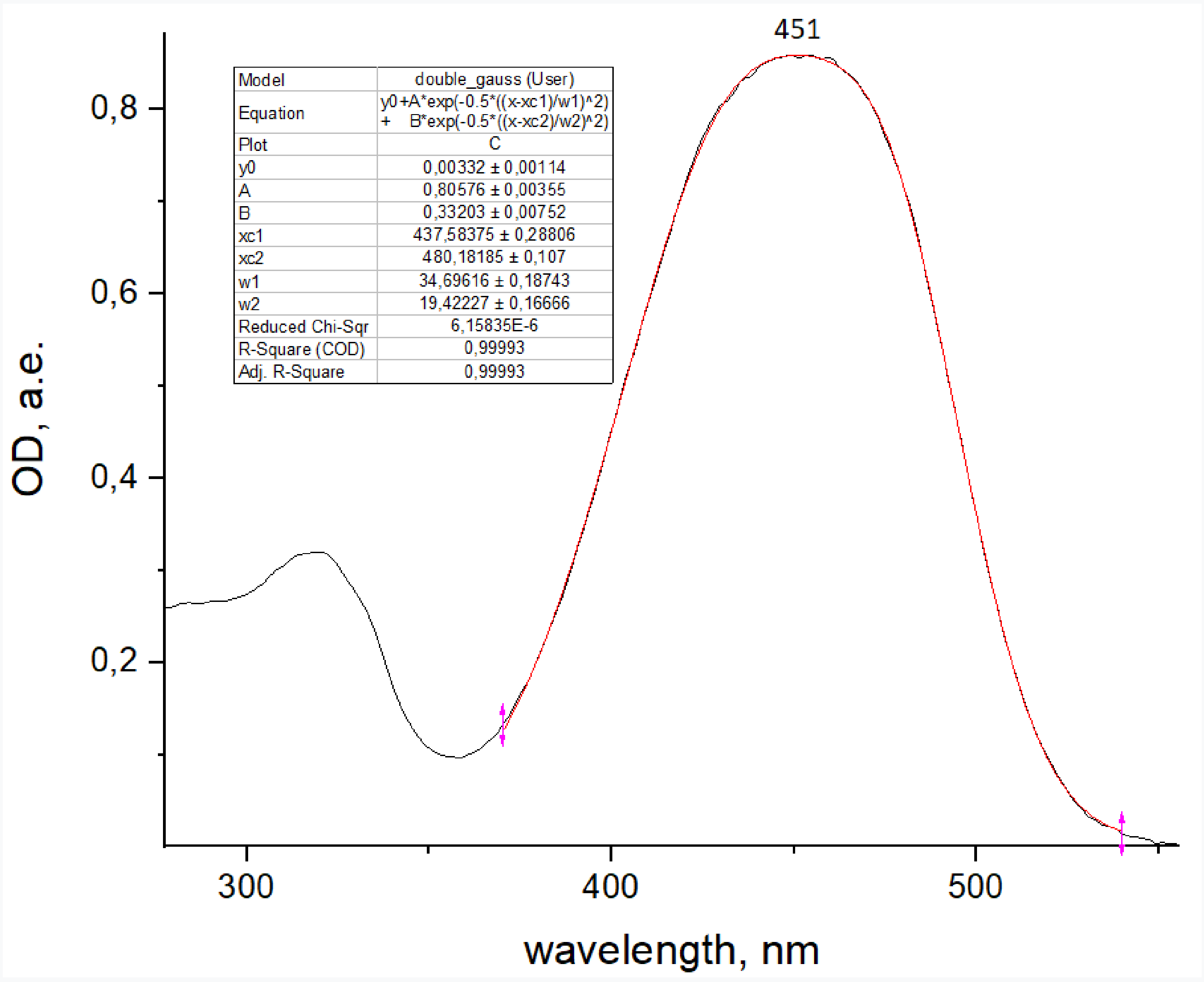

Figure S7. Decomposition of the absorption band of compound **2** (DENAQ) in DMSO into two Gaussian functions.

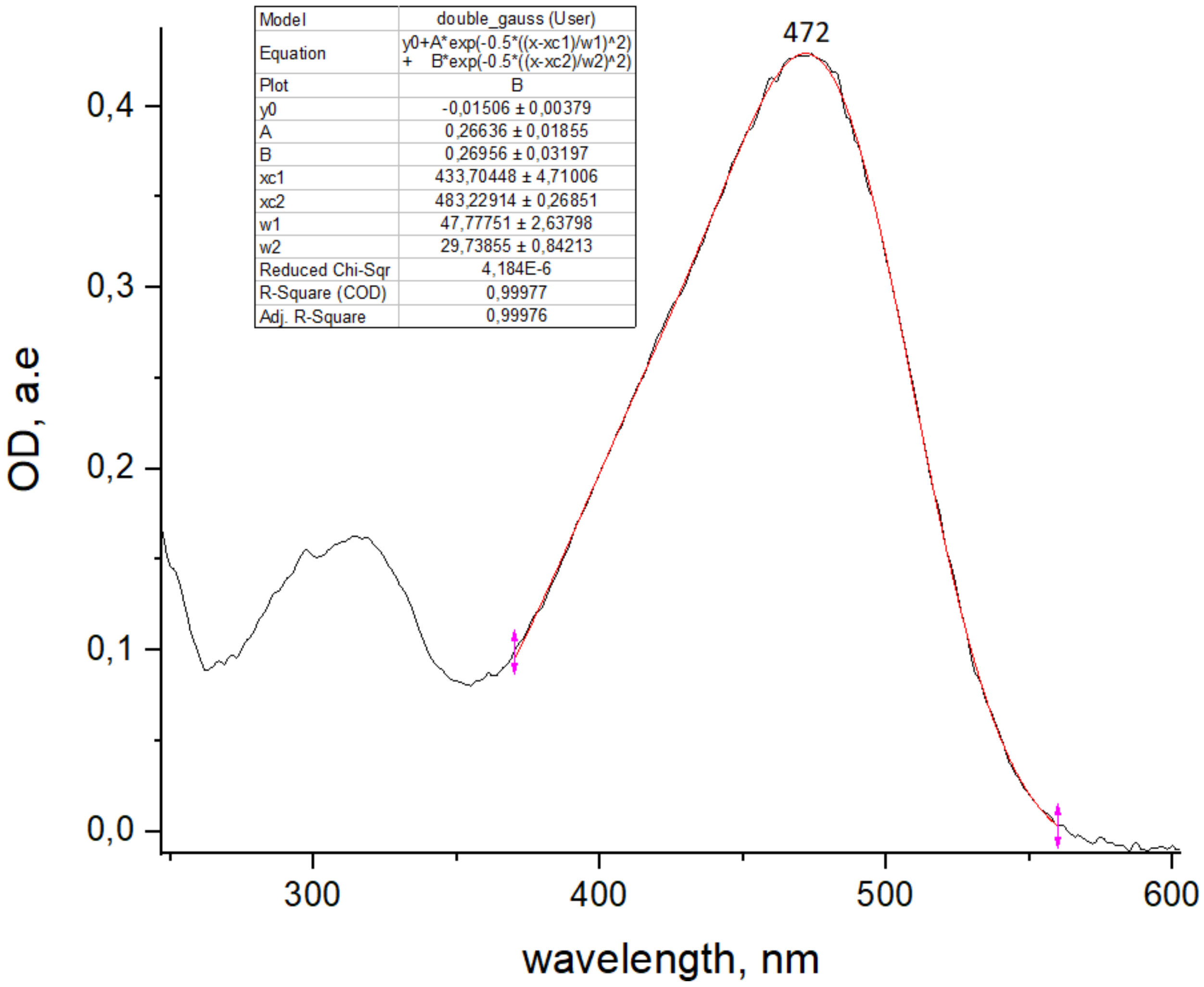

Figure S8. Decomposition of the absorption band of compound **2** (DENAQ) in water into two Gaussian functions.

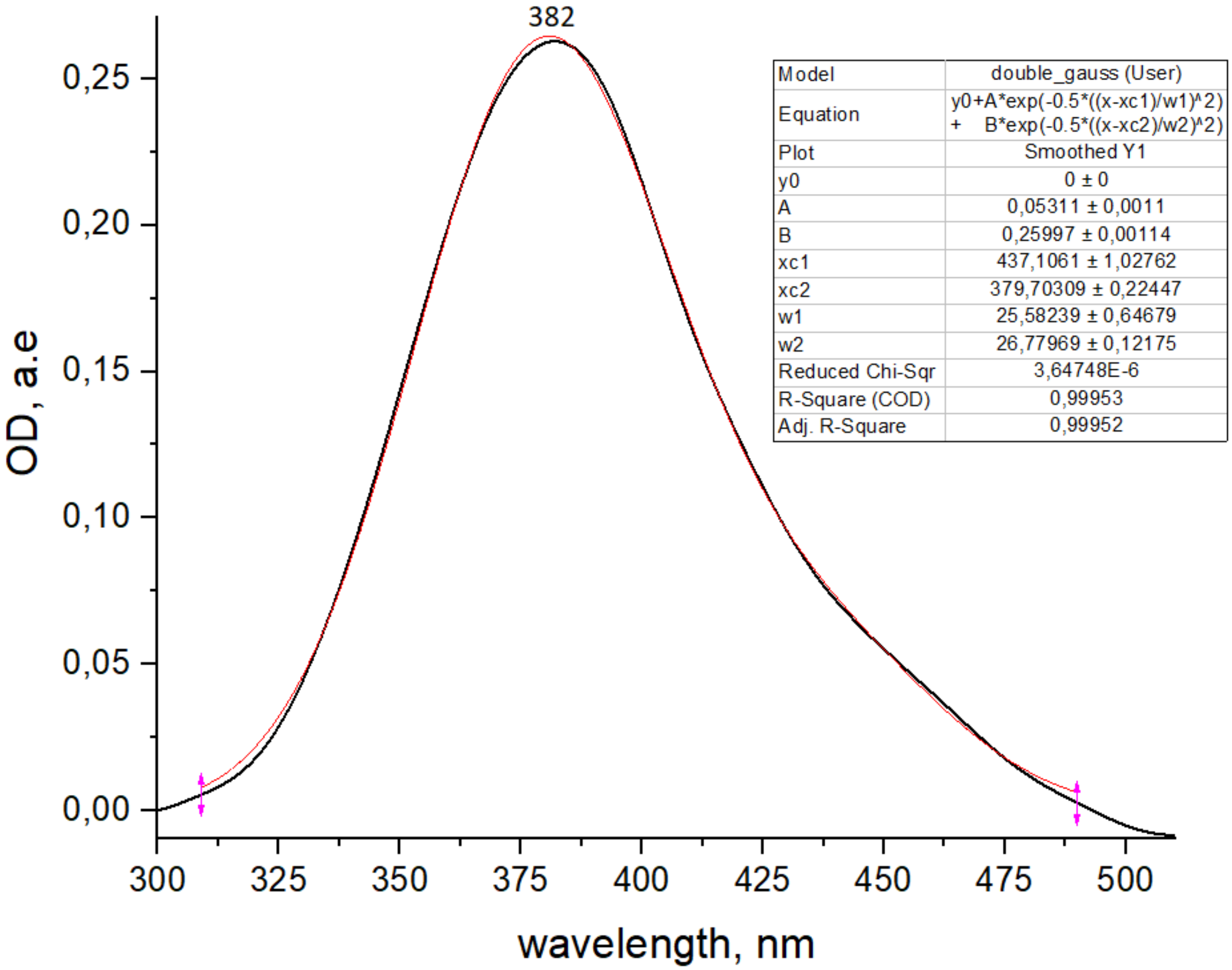

Figure S9. Decomposition of the absorption band of compound **6** in DMSO into two Gaussian functions.

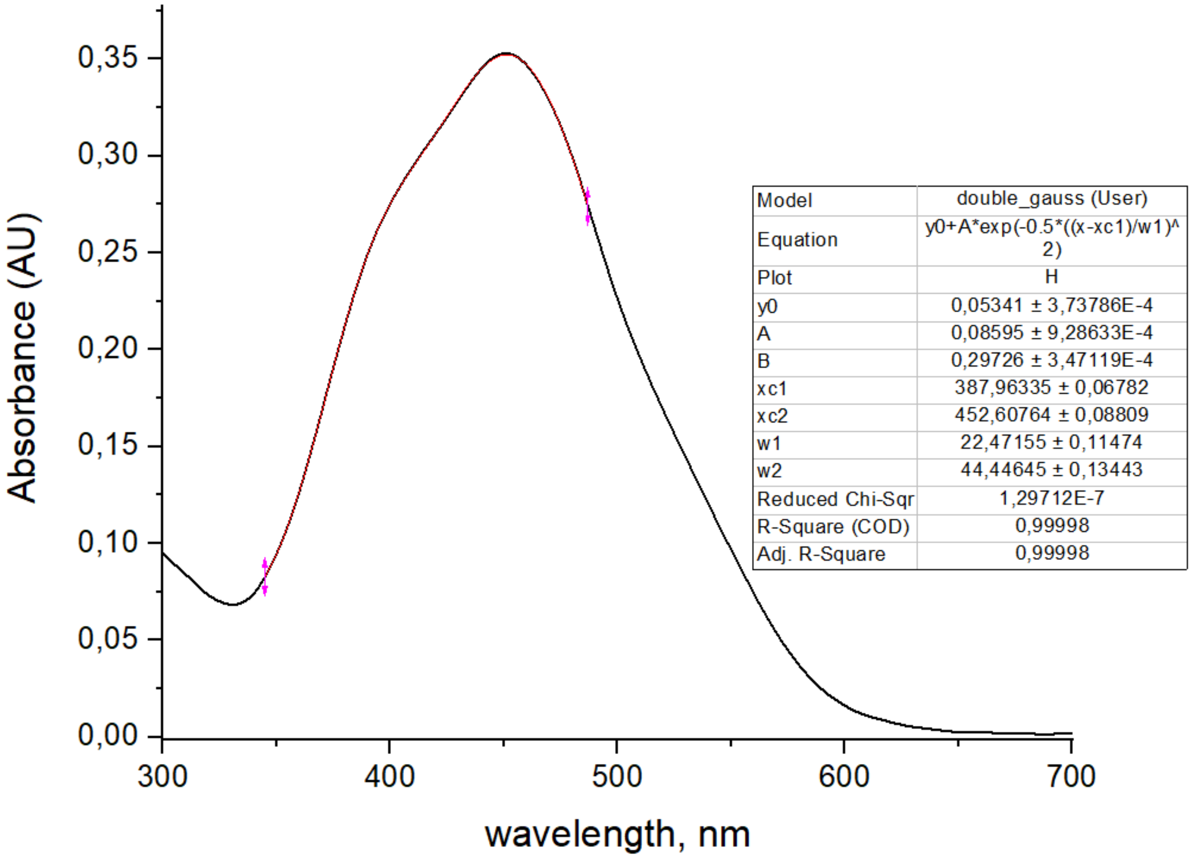

Figure S10. Decomposition of the absorption band of compound **7** in water into two Gaussian functions.

5. Supplementary Materials and methods

Preparation of APLs

Method A.

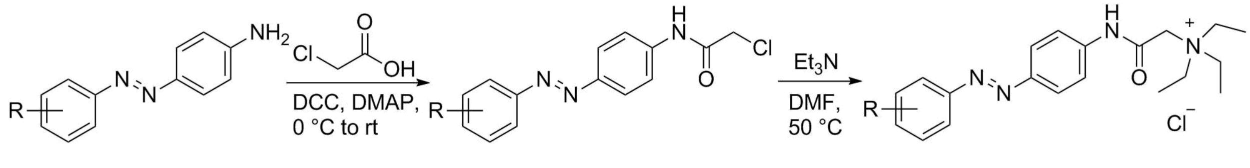

A two-step method for the preparation of APLs was described in the literature,[1] it includes the sequential acylation of the azoaniline derivatives with chloroacetic acid and coupling reagents or chloroacetyl chloride and base; and subsequent nucleophilic substitution of the chloride with triethylamine.

Method B.

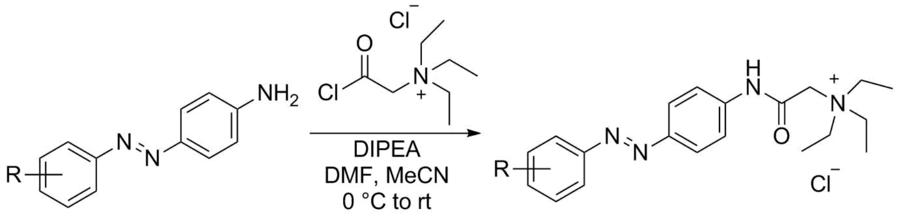

A one-step method of acylation of azoaniline derivatives with 2-triethylammonio acetic acid chloride chloride, the method was previously described in the literature.[2]

I. Preparation of
***N,N,N*-triethyl-2-oxo-2-((4-(phenyldiazenyl)phenyl)amino)ethan-1-aminium chloride (Ph-AQ, 1)**

1. Preparation of **4-(phenyldiazenyl)aniline** (**9**): To an ice-cooled solution of 5 ml of aniline in 50 ml of hydrochloric acid solution, 15 ml of sodium nitrite solution (3.784 g) was added dropwise within 40 minutes. Then the reaction mixture was stirred while cooling for another 40 min, after which it was stabilized with urea. To the resulting reaction mixture, a previously ice-cooled solution of 5 ml of aniline in water/HCl was added dropwise, after which pH of the solution was increased to 4 with NaOAc, and the mixture was stirred for another 2 hours. The crude product was extracted with ethyl acetate, the organic layer was washed with water, brine, and dried over Na_2SO_4, then the solvent was distilled off in vacuum. The product was isolated by flash chromatography using the mixture of PE-dichloromethane 5:1 as an eluent with the yield of 5.34 g (49%).

2. **2-chloro-*N*-(4-(phenyldiazenyl)phenyl)acetamide 10** was prepared from 0.4 g of compound **9**, 1.25 g (3 eq.) of DCC, 0.575 g (3 eq.) of chloroacetic acid and 0.03 g of DMAP with the yield of 0.316 g (57%) and used at next stage without further purification.

3. ***N,N,N*-triethyl-2-oxo-2-((4-(phenyldiazenyl)phenyl)amino)ethan-1-aminium chloride (1)** was prepared from 0.2 g of compound **10** and 0.204 mL (2 eq.) of triethylamine with the yield of 0.112 g (45%). ^{1}H NMR (DMSO-d6, 500 MHz) δ: 10.82 (1H, s), 8.06-7.85 (4H, m), 7.63-7.52 (4H, m), 7.41-7.30 (1H, m), 4.53 (2H, d, J = 3.0

Hz), 3.53 (6H, dd, J = 7.2 Hz, J = 4.6 Hz), 1.31 (9H, t, J = 7.2 Hz). HRMS (ESI–TOF): m/z calcd for $C_{20}H_{27}N_4O$ $[M]^+$: 339.2179; found 339.2181. This compound is known.

II. Preparation of DENAQ (2)

***N,N*-diethyl-4-((4-nitrophenyl)diazenyl)aniline 11** was prepared according to the literature procedure [3] from 2 g (14.5 mmol) of 4-nitroaniline 1, 1 g (14.5 mmol) $NaNO_2$ and 2.16 g (14.5 mmol) of *N,N*-diethylaniline. The product was recrystallized from ethanol and used in the next step without further purification. The product yield was 1.86 g (43%).

4-((4-aminophenyl)diazenyl)-*N,N*-diethylaniline 12 was prepared according to the literature procedure [from 1.5 g (5 mmol) of compound 2 and 2.4 g (10 mmol) of Na2S·9H2O with the yield of 0.735 g (55%). ^{1}H NMR (DMSO-d6, 500 MHz) δ: 7.67-7.61 (2H, m), 7.58-7.53 (2H, m), 6.76-6.69 (2H, m), 6.67-6.61 (2H, m), 5.77 (2H, br. s.), 3.40 (4H, q, *J* = 7.0 Hz), 1.12 (6H, t, *J* = 7.0 Hz). ^{13}C NMR ($CDCl_3$) δ: 150.9, 148.6, 143.3, 142.4, 123.8, 113.5, 111.0, 43.9, 12.5.

2-Chloro-*N*-(4-((4-(diethylamino)phenyl)diazenyl)phenyl)acetamide 13. To the solution of 268 mg (1 mmol) of compound **3**, 284 mg (3 mmol) of chloroacetic acid and 18 mg (0.15 mmol) of DMAP in dichloromethane was slowly added solution of 310 mg (1.5 mmol) DCC in dichloromethane. The reaction mixture was cooled in an ice bath while adding. Then the mixture was stirred for 2 hours at room temperature. Formed DCU was filtered, then the solvent was distilled. The reaction products were dissolved in 1,4-dioxane, the insoluble precipitate was filtered, and dioxane was distilled off. The products were purified by the method of column chromatography on silica gel (DCM to DCM:EtOAc 4:1). The product yield was 175 mg (68%). ^{1}H NMR ($CDCl_3$, 500 MHz) δ: 8.35 (1H, br.s.), 7.89-7.81 (4H, m), 7.69-7.60 (2H, m), 6.73 (2H, d, J = 8.8Hz), 4.21 (2H, s), 3.41 (4H, q, J = 7.1 Hz), 1.23 (6H, t, J = 7.1 Hz). ^{13}C NMR ($CDCl_3$) δ: 163.6, 150.3, 137.4, 125.3, 123.0, 120.1, 119.5, 111.1, 44.8, 42.9, 12.6. HRMS (ESI–TOF): m/z calcd for $C_{18}H_{22}ClN_4O$ $[M+H]^+$ 345.1477; found 345.1477.

2-((4-((4-(diethylamino)phenyl)diazenyl)phenyl)amino)-*N,N,N*-triethyl-2-oxoethan-1-aminium chloride (DENAQ) 2. To the mixture of 185 mg (0.25 mmol) of compound **3** in 10 mL of DMF was slowly added 0.3 mL of Et_3N in 3 mL of DMF. Reaction mixture was heated to 50 °C and stirred overnight at this temperature. The solvent was reduced in vacuum, reaction products were purified with column chromatography (acetone - acetone:MeOH 1:10). The initial compound was isolated and reused. The yield was 93 mg (39%) ^{1}H NMR (DMSO, 500 MHz) δ: 8.26-7.82 (8H, m), 4.60-4.56 (2H, m), 3.57 (6H, q, *J* = 7.2 Hz), 3.51 (4H, q, *J* = 7.2 Hz), 1.39 (9H, t, *J* = 7.2 Hz), 1.22 (6H, t, *J* = 7.2 Hz). HRMS (ESI–TOF): m/z calcd for $C_{24}H_{36}N_5O^+$ 410.2914; found 410.2914. UV/Vis (H_2O): λ_{max} = 473 nm. This compound is known.

III. Preparation of *N,N,N*-triethyl-2-oxo-2-((4-(p-tolyldiazenyl)phenyl)amino)ethan-1-aminium chloride (3).

1. 4-(p-Tolyldiazenyl)aniline (16) was obtained according to the same procedure for compound **11** from 2 g of p-toluidine, 1.29 g of $NaNO_2$ and 1.732 mL of aniline. The crude product was isolated by flash chromatography (silica gel, PE/EtOAC 5:1) and used in the next step. ^{1}H NMR (DMSO-d6, 500 MHz) δ: 12.14 (1H, br. s), 8.95 (1H, br. s), 7.96-7.91 (2H, m), 7.86-7.77 (4H, m), 7.42-7.36 (2H, m), 2.40 (3H, s). ^{13}C NMR (DMSO-d6, 125 MHz) δ: 153.5, 150.0, 149.3, 141.7, 130.0, 123.8, 122.5, 119.6, 21.0.

2. ***N,N,N*-triethyl-2-oxo-2-((4-(p-tolyldiazenyl)phenyl)amino)ethan-1-aminium chloride (3)** was prepared according to the literature procedure [2] (scheme B) from 0.568 g of **16**, 1.417 g of betaine **14** and 1.25 mL of oxalyl chloride. The yield of compound **5** after purification was 137 mg (13%). ^{1}H NMR (DMSO-d6, 500 MHz) δ: 8.87 (1H, s), 8.00-7.94 (1H, m), 7.91-7.89 (1H, m), 7.83-7.77 (2H, m), 7.77-7.70 (2H, m), 7.44-7.38 (2H, m), 3.60-3.48 (6H, m), 3.40 (2H, s), 2.41 (3H, s), 1.30 (9H, t, J = 8.3 Hz). ^{13}C NMR (DMSO-d6, 125 MHz) δ: 162.4, 150.1, 148.2, 141.5, 129.9, 129.4, 122.4, 120.0, 119.5, 59.5, 54.7, 21.0, 7.6. HRMS (ESI): m/z calcd for C21H29N4O [M]+: 353.2336; found 353.2333.

IV. Preparation of 2-((4-((4-chlorophenyl)diazenyl)phenyl)amino)-*N*,*N*,*N*-triethyl-2-oxoethan-1-aminium chloride (4).

1. **(Carboxymethyl)triethylammonium chloride (14)**. This compound was obtained according to the literature procedure [4] in two stages from 10 mL of methyl chloroacetate and 32 mL of triethylamine with the yield of 13.36 g (60% on 2 stages). ^{1}H NMR (DMSO-d6, 500 MHz) δ: 4.28 (2H, s), 3.50 (6H, q, *J* = 7.2 Hz), 1.20 (9H, t, *J* = 7.2 Hz). ^{13}C NMR (DMSO-d6, 125 MHz) δ: 166.2, 55.1, 53.7, 7.5.

2. Preparation of **4-((4-chlorophenyl)diazenyl)aniline (15).** Compound 17 was obtained according to the general procedure of azo coupling from 0.92 g of 4-chloroaniline, 0.5 g of $NaNO_2$ and 0.66 mL of aniline in water/acetonitrile solution with the yield of 0.26 g.

3. **2-((4-((4-chlorophenyl)diazenyl)phenyl)amino)-*N*,*N*,*N*-triethyl-2-oxoethan-1-aminium chloride (4)** was prepared according to the literature procedure from 0.2 g of compound **15**, 0.55 g of betaine **14** and $(COCl)_2$. The yield of the compound after purification was 0.056 g (16%). ^{1}H NMR (DMSO-d6, 500 MHz) δ: 7.95-7.84 (6H, m), 7.67-7.61 (2H, m), 4.46 (2H, s), 3.58-3.48 (6H, m), 1.30 (9H, t, *J* = 7.2 Hz). ^{13}C NMR (DMSO-d6, 125 MHz) δ: 162.5, 150.6, 148.1, 129.6, 128.5, 124.1, 124.0, 123.8, 120.1, 56.5, 54.2, 7.6. HRMS (ESI): m/z calcd for $C_{20}H_{26}ClN_4O$ $[M]^+$: 373.1790; found 373.1790.

V. Preparation of *N*,*N*,*N*-triethyl-2-((4-((4-nitrophenyl)diazenyl)phenyl)amino)-2-oxoethan-1-aminium chloride (5).

Compound **7** was obtained according to the general **procedure A** from 1eq. of **2-chloro-N-(4-((4-nitrophenyl)diazenyl)phenyl)acetamide** and 1.5 eq. of triethylamine with the yield of 67%. ^{1}H NMR (DMSO-d6, 500 MHz) δ: 11.66 (1H, s), 8.48-8.39 (2H, m), 8.09-8.05 (2H, m), 8.04-8.00 (2H, m), 7.95-7.92 (2H, m), 4.37 (2H, s), 3.55 (6H, q, J = 7.2 Hz), 1.30 (9H, t, J = 7.2 Hz). ^{13}C NMR (DMSO-d6, 125 MHz) δ: 162.6, 155.2,

148.3, 148.2, 142.0, 125.1, 124.5, 123.3, 120.1, 56.4, 54.2, 7.5. HRMS (ESI): m/z calcd for $C_{20}H_{26}N_5O_3^+$: 384.2030, found: 384.2032.

VI. Preparation of *N,N,N*-triethyl-2-((4-((4-hydroxyphenyl)diazenyl)phenyl)amino)-2-oxoethan-1-aminium chloride (6).

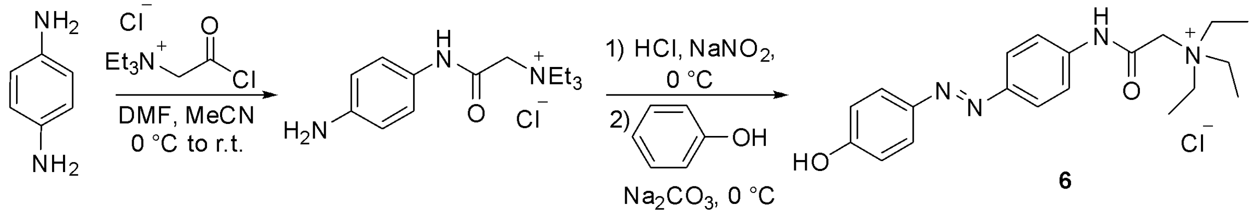

Scheme of synthesis of compound 4

1) Preparation of **2-((4-aminophenyl)amino)-N,N,N-triethyl-2-oxoethan-1-aminium chloride (17)**. To the ice-cooled solution of 1g of p-phenylenediamine and 4 mL of DIPEA in 20 mL of DMF was added dropwise solution of **2-chloro-*N,N,N*-triethyl-2-oxoethan-1-aminium chloride**, prepared according to the literature procedure [2] from 2.7 g of **14** and $(COCl)_2$, in 15 mL of DMF/acetonitrile. The mixture was stirred for 1 hour, then the formed precipitate was filtered off, and the solvent was distilled off in vacuum. The crude product was loaded onto silica gel, and was eluted with acetone, then with water.

2) ***N,N,N*-triethyl-2-((4-((4-hydroxyphenyl)diazenyl)phenyl)amino)-2-oxoethan-1-aminium (6)**. To a solution of 0.5 g of **15** in 10 ml of water, a solution of hydrochloric acid was added dropwise to pH 2, then the solution was cooled with ice. To the mixture was added dropwise a solution of 0.12 g of $NaNO_2$ in 3 mL of water, after which the mixture was stirred for 1 h. The resulting diazonium salt was then added dropwise to an ice-cold water solution of 0.165 g of phenol with pH=10. The pH during the addition of diazonium salt was stabilized with Na_2CO_3. After adding the entire volume, the mixture was stirred for an hour, after which was neutralized with a HCl solution. The water was distilled in a vacuum, then the crude product was dissolved in acetonitrile, the precipitate was filtered

off, after which acetonitrile was distilled in a vacuum. From the obtained residue the product was isolated by flash chromatography (silica gel, acetone-acetone/water 1:3). 10.23 (1H, s), 9.97 (1H, s), 7.98-7.72 (5H, m), 7.03-6.86 (3H, m), 4.25 (2H, s), 3.63-3.51 (6H, m), 1.30 (9H, t, J = 6.6 Hz). HRMS (ESI–TOF): m/z calcd for $C_{20}H_{27}N_4O_2$ $[M]^+$: 355.2129; found 355.2119.

VII. Preparation of *N*-(4-(phenyldiazenyl)phenyl)acetamide (8).

To an ice-cooled solution of 0.49 g (1 eq.) of 4-aminoazobenzene **9**, 0.425 mL (3 eq.) of glacial acetic acid and 0.05 g of DMAP in 50 mL of dry DCM was added dropwise solution of 1.03 g (2 eq.) of DCC in 15 mL of DCM during 10 min. The resulting mixture was stirred with cooling for an additional 15 minutes, then at room temperature for 1 hour. The formed precipitate was filtered off, then the solvent was distilled off in vacuum. The resulting crude product was dissolved in 50 ml of 1,4-dioxane, then the mixture was cooled, and the resulting precipitate was also filtered off. The product was isolated by flash chromatography using the mixture of PE-ethyl acetate 5:1 to 1:2 as an eluent with the yield of 0.52 g (76%). ^{1}H NMR ($CDCl_3$, 500 MHz) δ: 7.94-7.87 (4H, m), 7.73 (1H, br. s.), 7.72-7.65 (2H, m), 7.54-7.44 (3H, m), 2.21 (3H, s). ^{13}C NMR ($CDCl_3$, 125 MHz) δ: 168.6, 152.7, 149.0, 140.5, 130.7, 129.0, 123.9, 122.7, 119.7, 24.7. HRMS (ESI): m/z calcd for $C_{14}H_{14}N_3O$ $[M+H]^+$: 240.1131; found 240.1132.

Spectral copies of 1H and 13C NMR of compounds

^{1}H NMR (400MHz, DMSO-d6) spectrum for 1

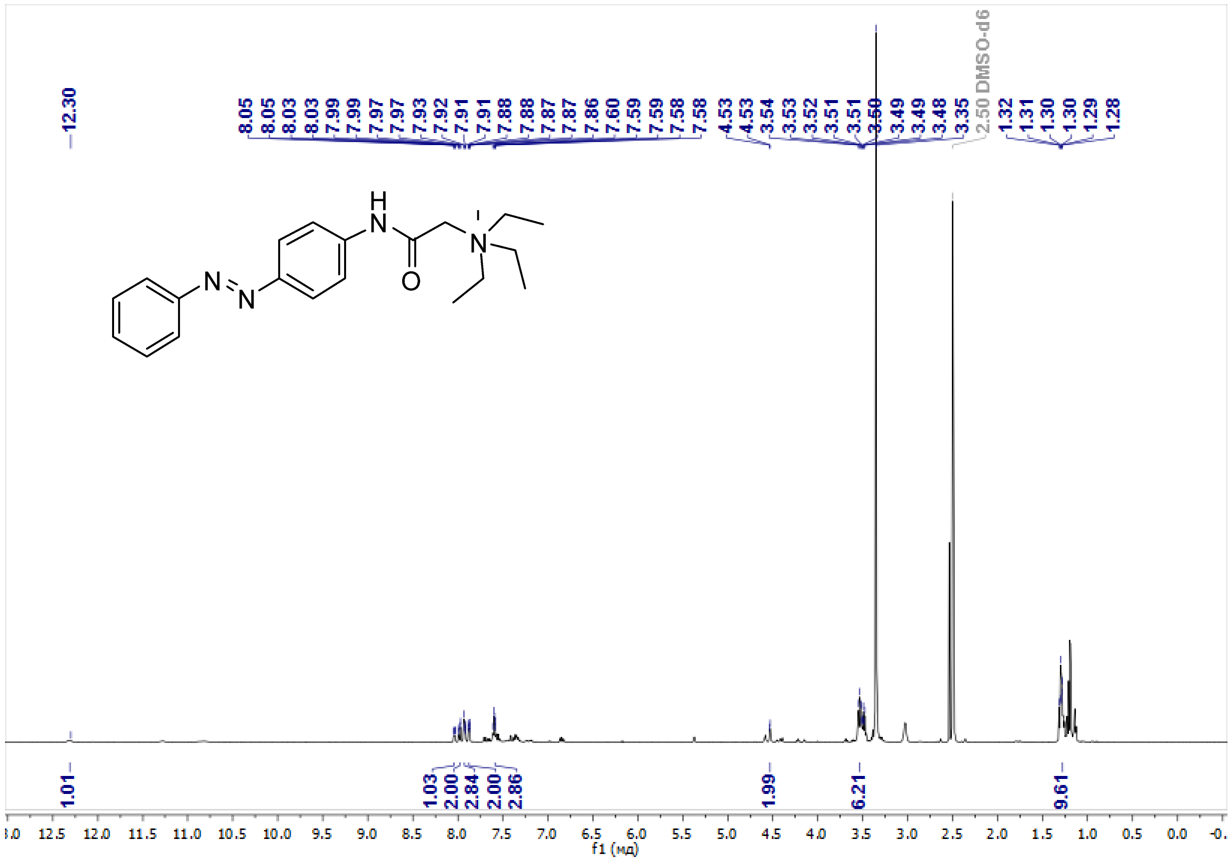

Region of 1H NMR spectrum for 1 that shows the presence of both *E* and Z isomers.

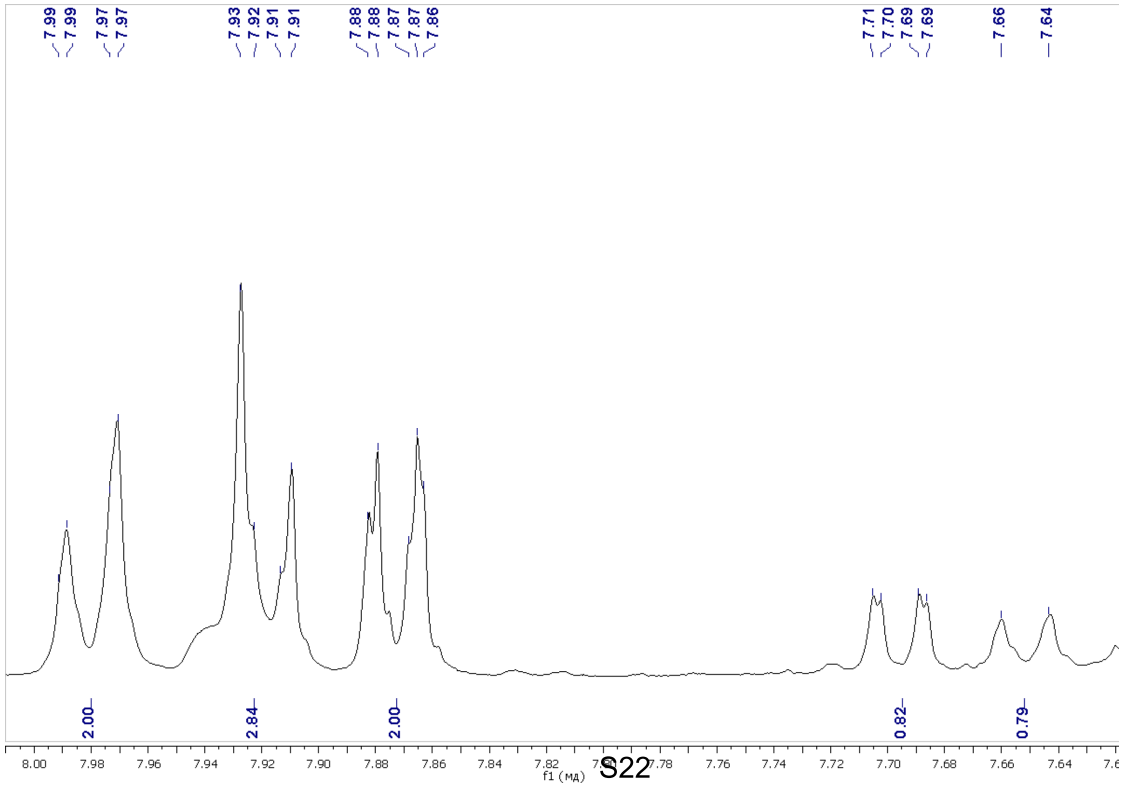

^{1}H NMR (400MHz, $CDCl_3$) spectrum for 13

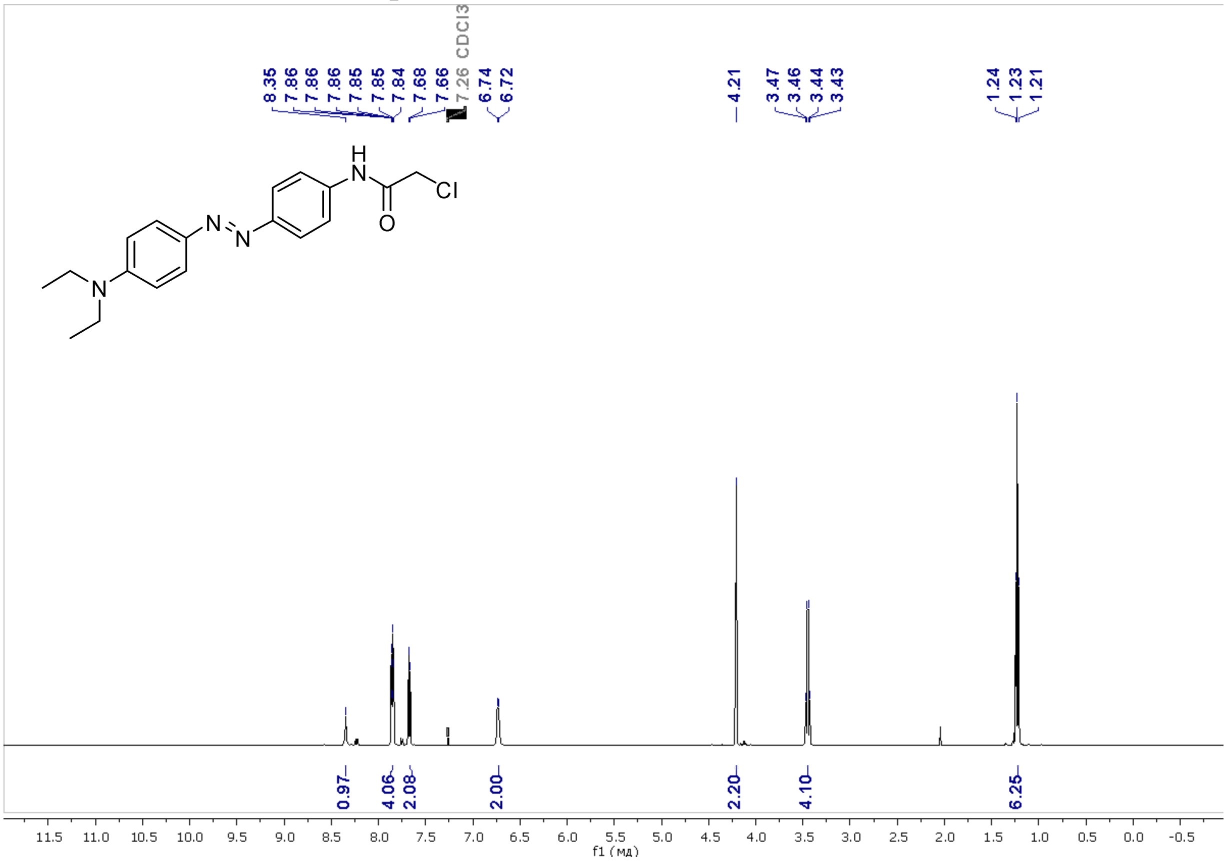

^{13}C NMR (101MHz, $CDCl_3$) spectrum for 13

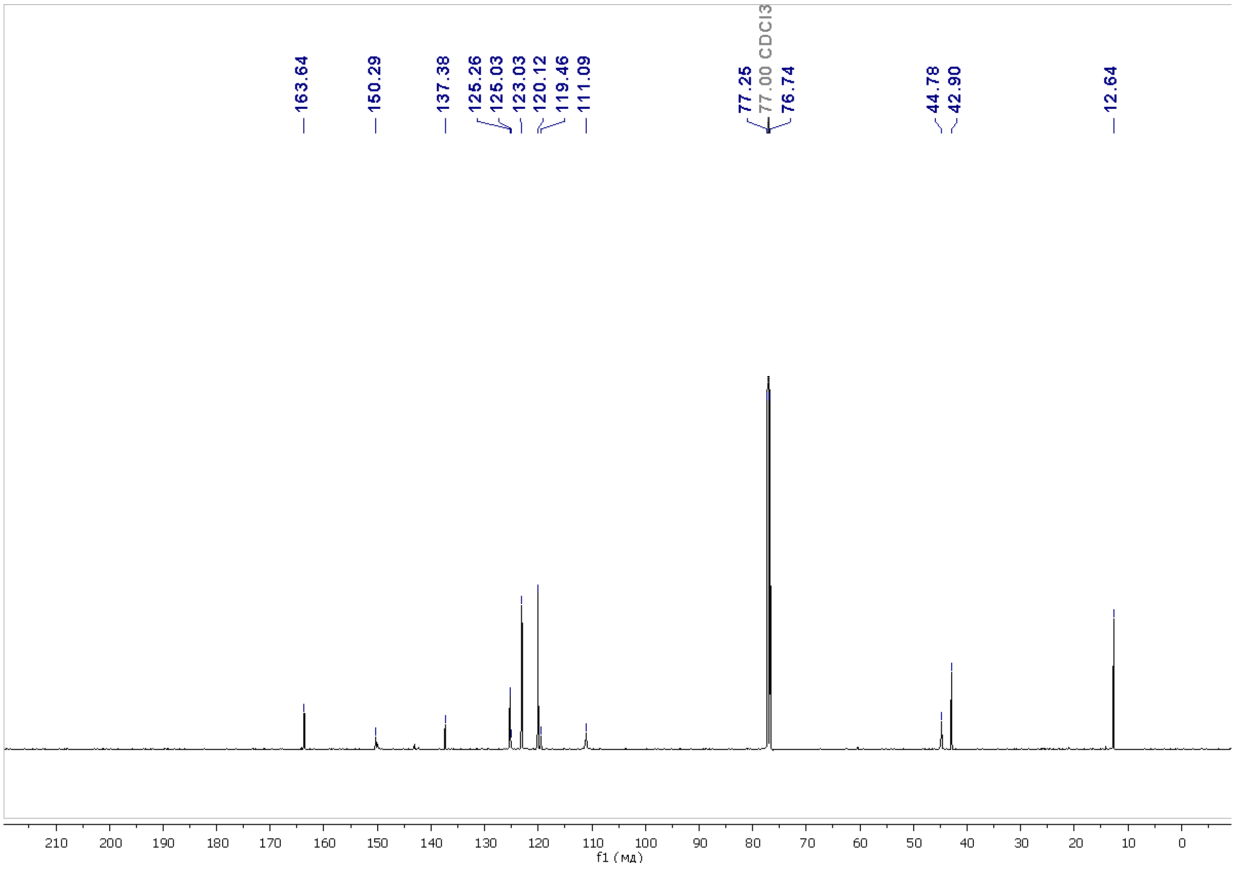

^{1}H NMR (500MHz, DMSO-d6) spectrum for 3

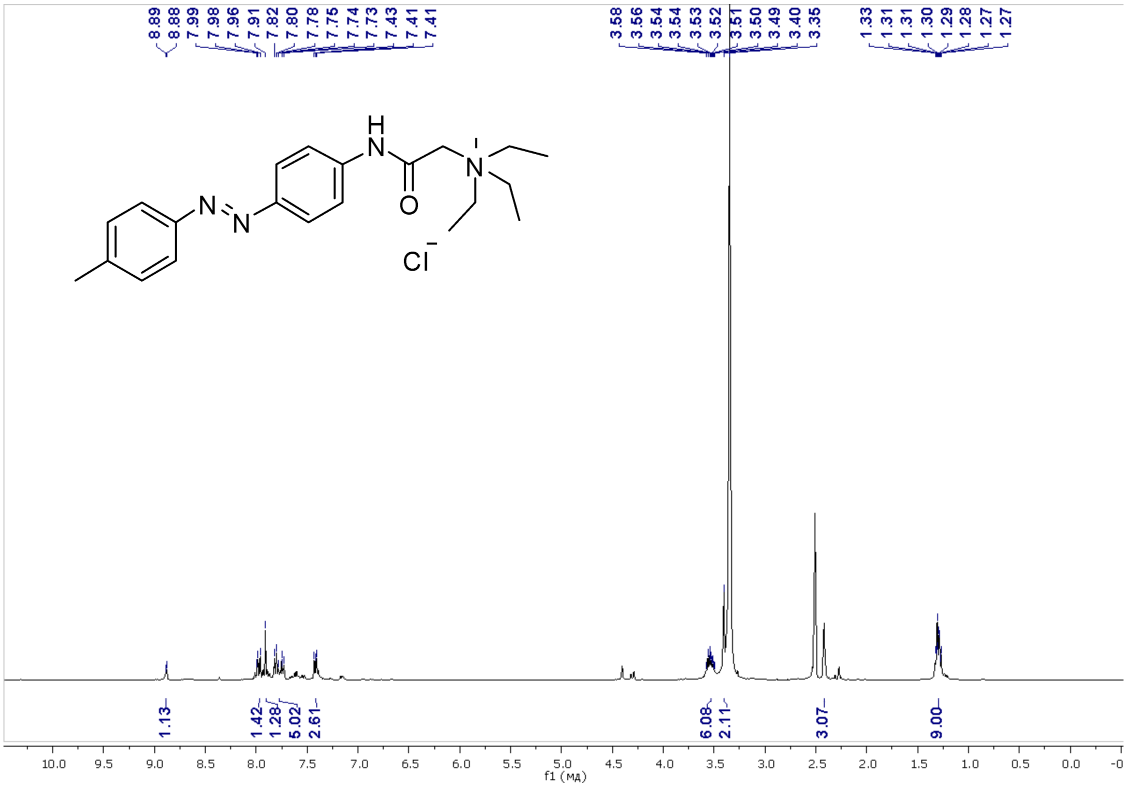

Region of 1H NMR spectrum for 3 that shows the presence of both *E* and *Z* isomers.

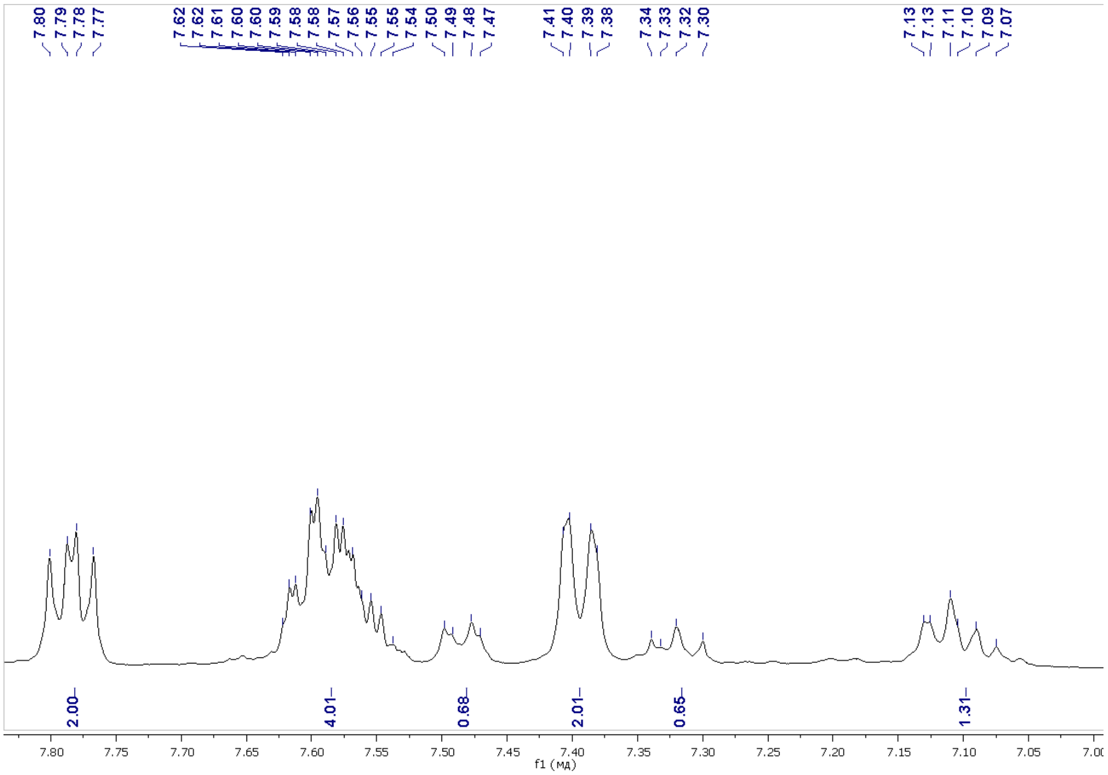

^{1}H NMR (500MHz, DMSO-d6) spectrum for 4

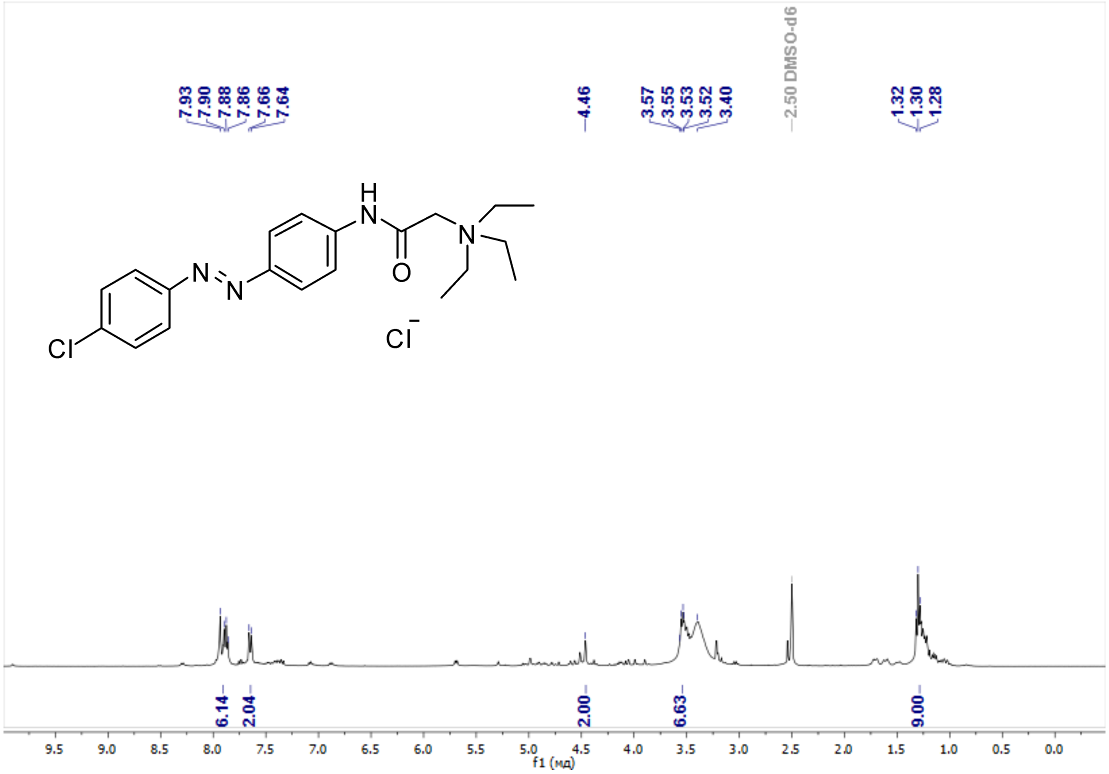

Region of 1H NMR spectrum for 4 that shows the presence of both *E* and Z isomers.

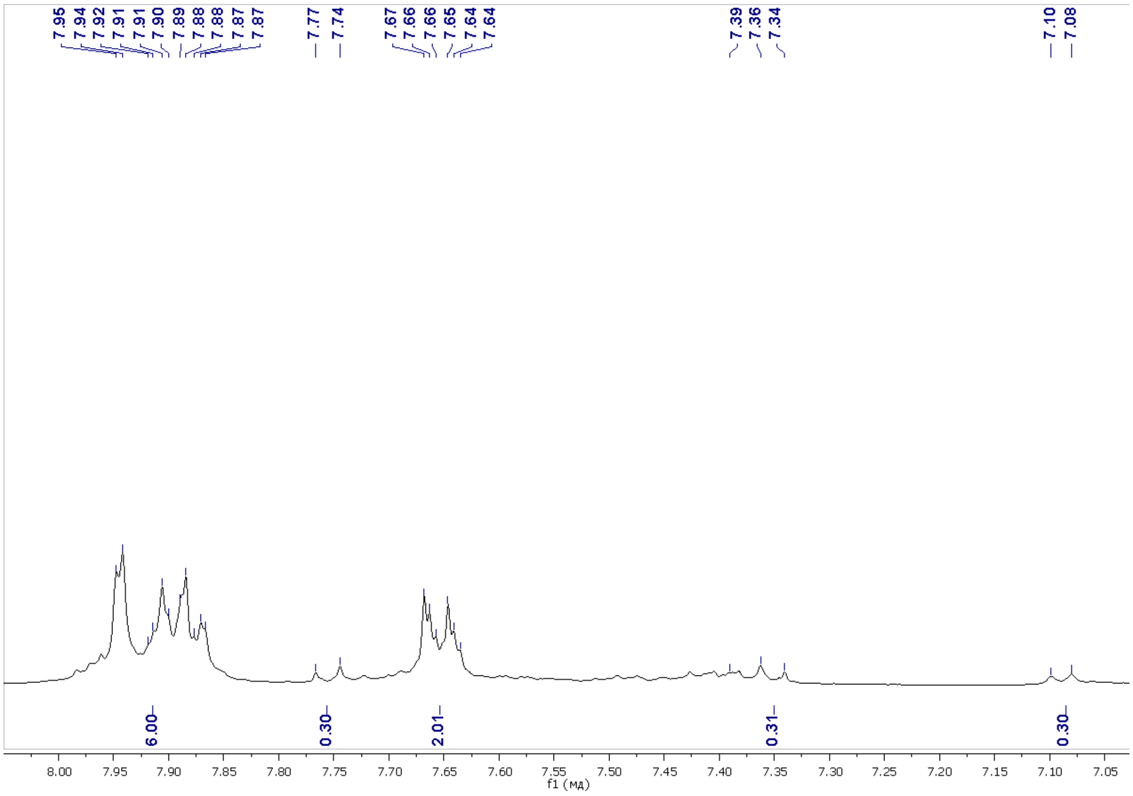

^{1}H NMR (500MHz, DMSO-d6) spectrum for 5

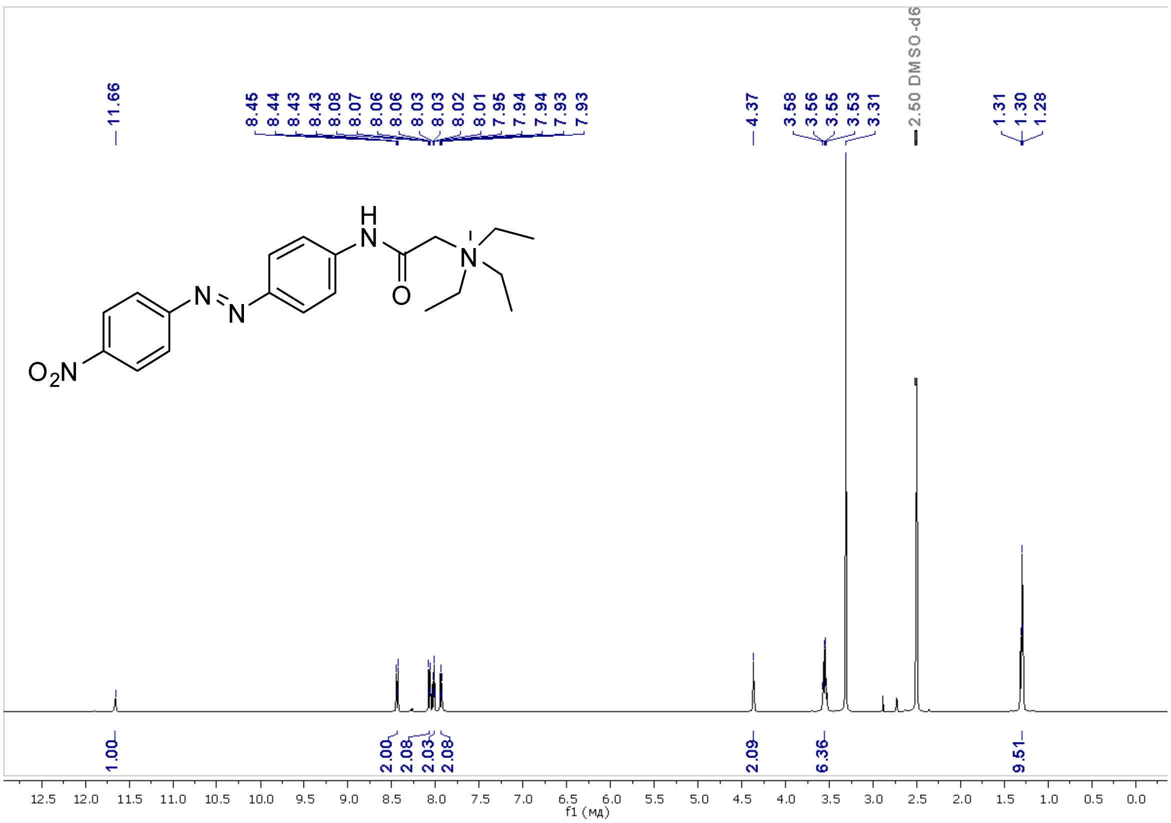

^{13}C NMR (125MHz, DMSO-d6) spectrum for 5

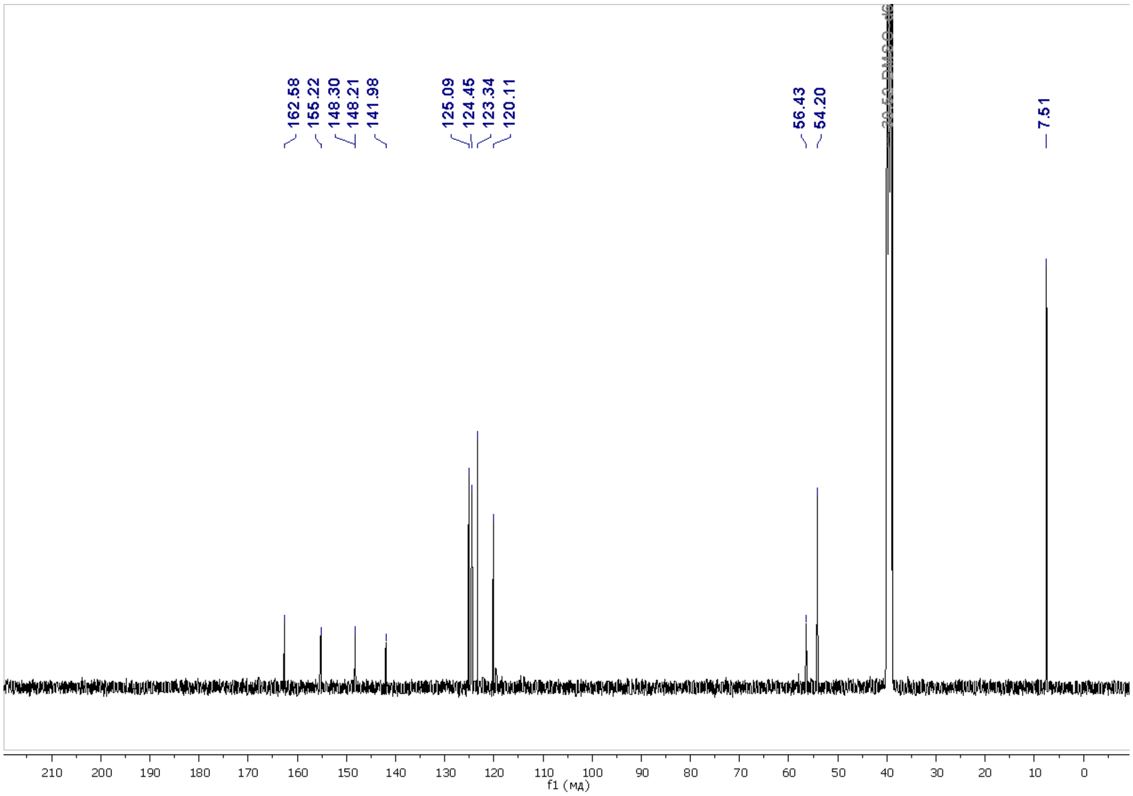

162.58
155.22
148.30
148.21
141.98
125.09
124.45
123.34
120.11
56.43
54.20
7.51
210 200 190 180 170 160 150 140 130 120 110 100 90 80 70 60 50 40 30 20 10 0
f1 (мд)

Region of 1H NMR spectrum for 5 that shows the presence of both *E* and Z isomers.

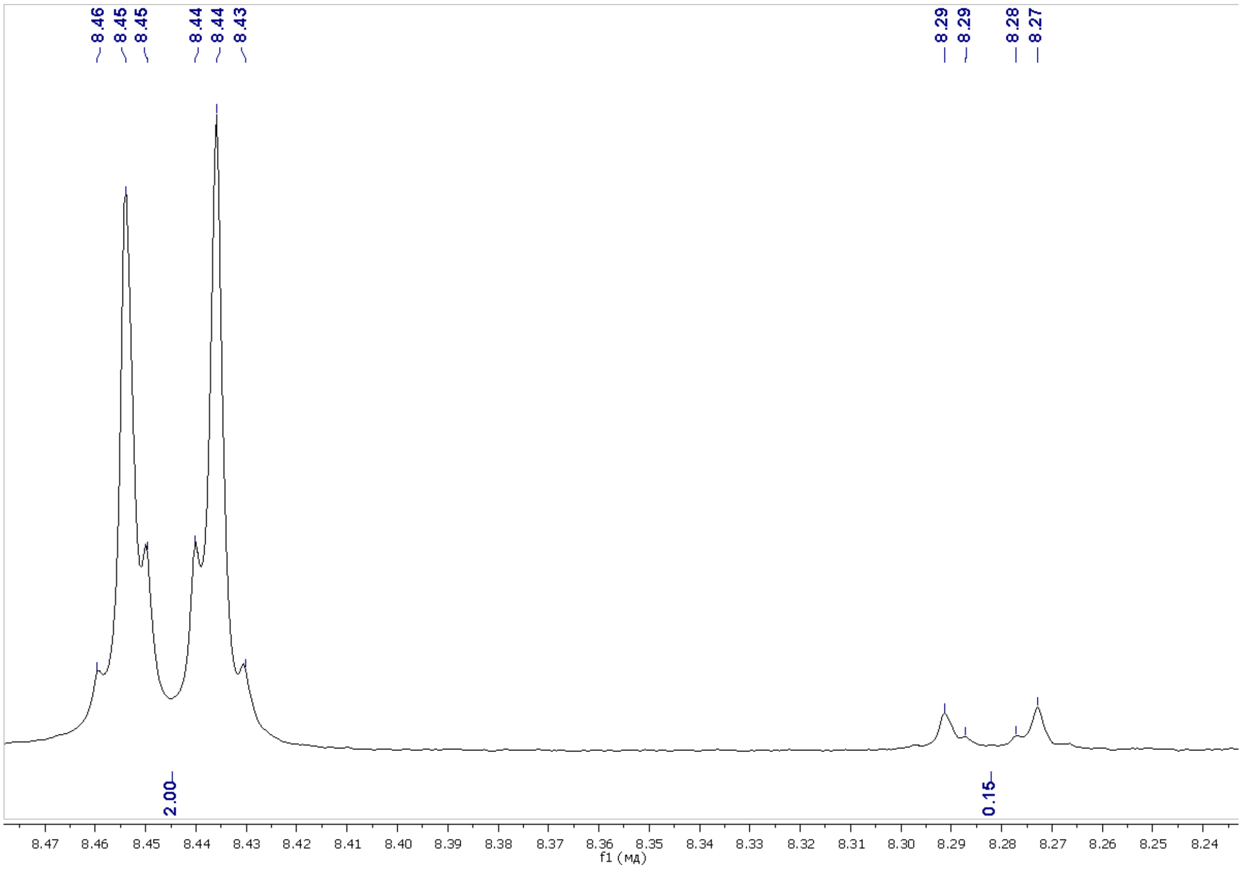

^{1}H NMR (500MHz, $CDCl_3$) spectrum for 8

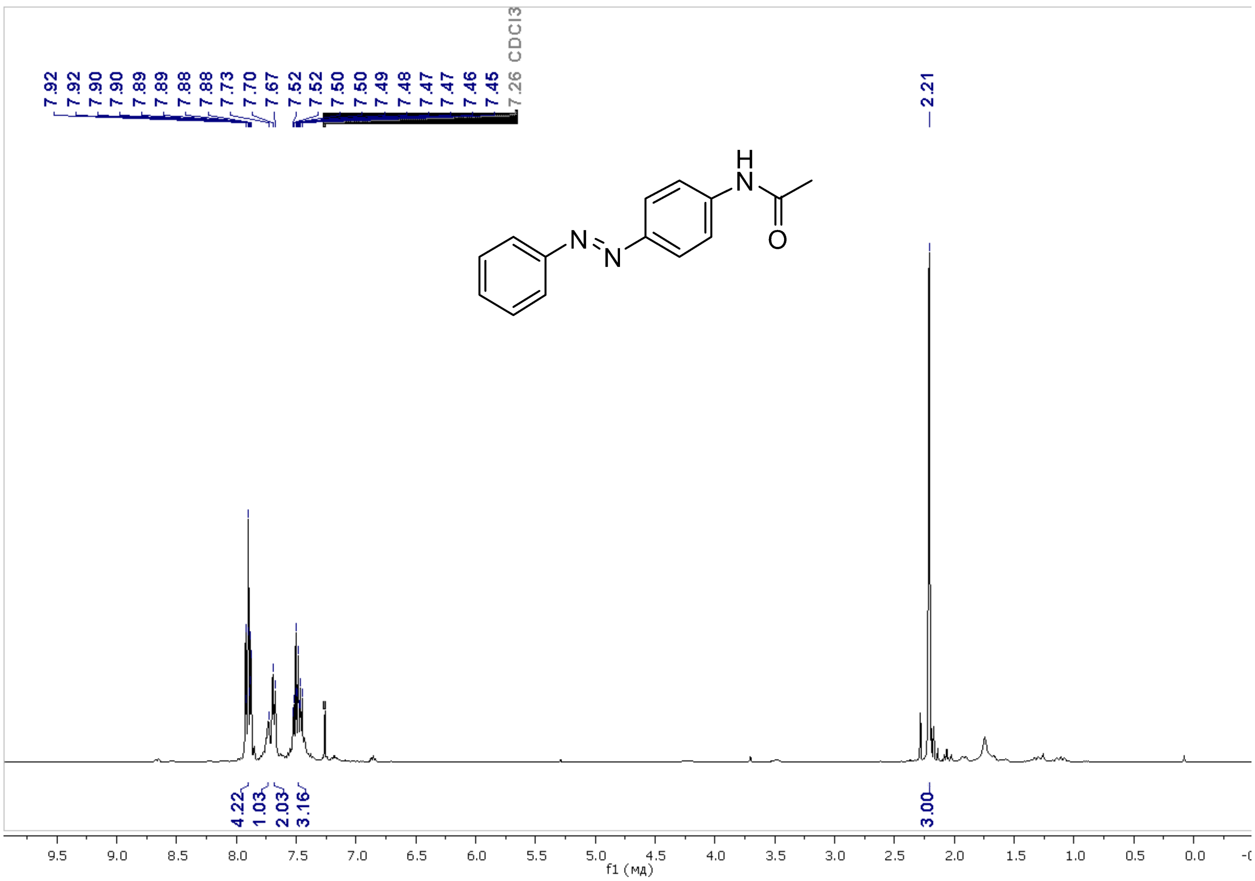

^{13}C NMR (125MHz, CDCl$_3$) spectrum for 8

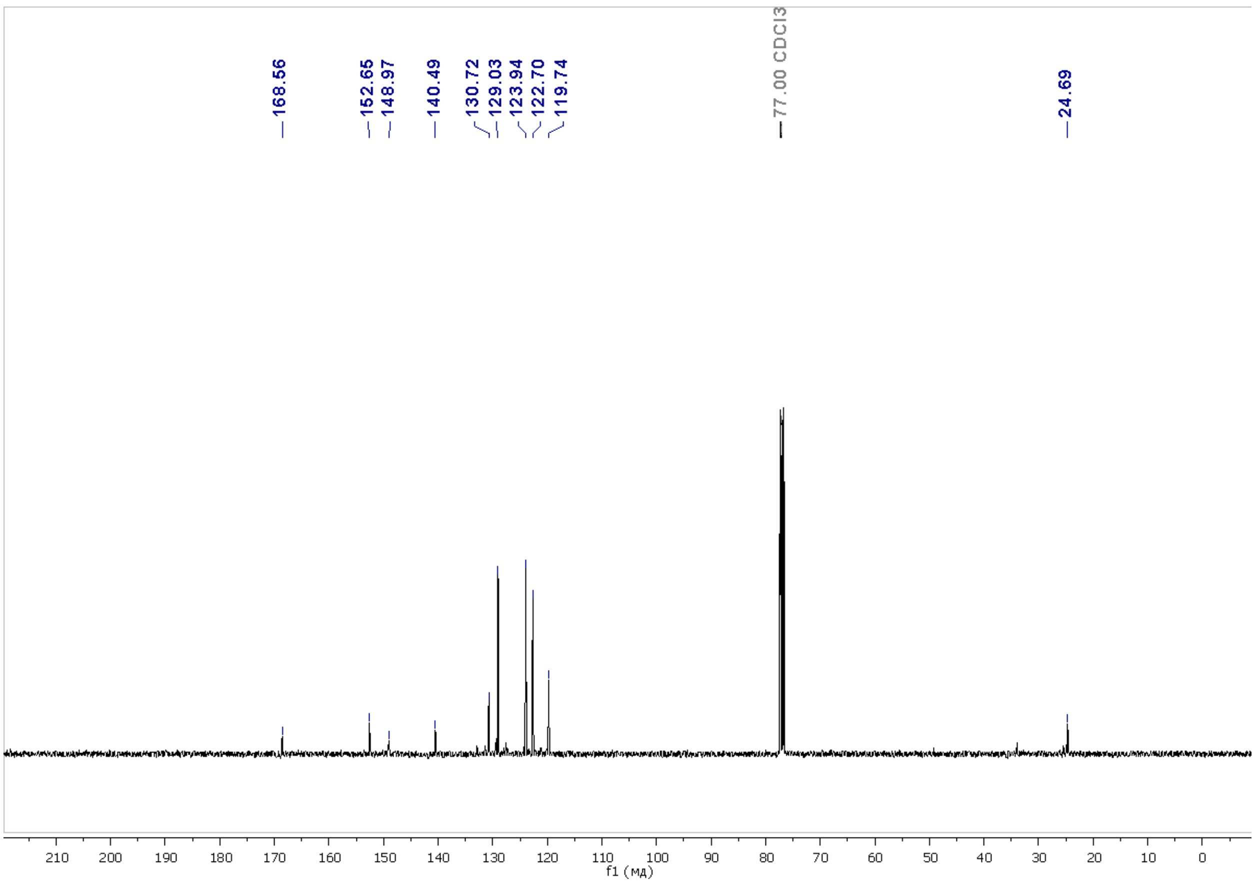

References

1. A. Y. Rotov, L. Astakhova, V. Sitnikova, A. Evdokimov,V. Boitsov, M. Dubina, M. R. and M. F. New experimental models of retinal degeneration for screening molecular photochromic ion channel blockers. (2018). Available at: https://cyberleninka.ru/article/n/new-experimental-models-of-retinal-degeneration-for-screening-molecular-photochromic-ion-channel-blockers/viewer. (Accessed: 6th May 2020)

2. Fortin, D. L. *et al.* Photochemical control of endogenous ion channels and cellular excitability. *Nat. Methods* **5**, 331–338 (2008).

3. Gao, D., Yang, D., Cui, H., Huang, T. & Lin, J. Synthesis and Measurement of Solubilities of Reactive Disperse Dyes for Dyeing Cotton Fabrics in Supercritical Carbon Dioxide. *Ind. Eng. Chem. Res.* **53**, 13862–13870 (2014).

4. Challenger, F.,Taylor, P., Taylor, B. "13. The interaction of betaine with primary aromatic amines, organic disulphides, and sodium sulphite." Journal of the Chemical Society (Resumed) (1942): 48-55.
